# Nutritional Strategies to Improve Post-exercise Recovery and Subsequent Exercise Performance: A Narrative Review

**DOI:** 10.1007/s40279-025-02213-6

**Published:** 2025-04-12

**Authors:** Alireza Naderi, Jeffrey A. Rothschild, Heitor O. Santos, Amin Hamidvand, Majid S. Koozehchian, Abdolrahman Ghazzagh, Erfan Berjisian, Tim Podlogar

**Affiliations:** 1 Department of Sport Physiology, Faculty of Human Sciences, Borujerd Branch, Islamic Azad University, Borujerd, Iran; 2 Sports Performance Research Institute New Zealand, Auckland University of Technology, Auckland, New Zealand; 3 High Performance Sport New Zealand, Auckland, New Zealand; 4 Postgraduate Program, Faculdade UNIGUAÇU, Cascavel, PR Brazil; 5 Department of Biological Sciences in Sport, Faculty of Sport Sciences and Health, Shahid Beheshti University, Tehran, Iran; 6 Department of Kinesiology, Jacksonville State University, Jacksonville, AL 36265 USA; 7 Department of Sport Sciences, Faculty of Humanities, Tarbiat Modares University, Tehran, Iran; 8 School of Medical and Health Sciences, Edith Cowan University, Joondalup, Western Australia Australia; 9 School of Sport, Exercise and Rehabilitation Sciences, College of Life and Environmental Sciences, University of Birmingham, Birmingham, UK; 10 Department of Public Health and Sport Sciences, Medical School, University of Exeter, Exeter, UK

## Abstract

Post-exercise recovery strategies influence the body’s ability to restore physiological homeostasis, replenish energy stores, repair muscle damage, and promote desired adaptations, which improve exercise performance. This narrative review examines the impact of nutritional strategies commonly used for enhancing recovery and subsequent exercise performance, particularly when athletes face short recovery periods. Carbohydrate ingestion is essential for glycogen replenishment, especially within the initial hours post-exercise, with its impact dependent on the types, timing, and amount. Protein is essential for accelerating muscle recovery and achieving a positive nitrogen balance, depending on the type and dosage. The co-ingestion of carbohydrates with proteins or fats is explored for its role in maximizing glycogen resynthesis and muscle repair, with evidence supporting the addition of protein to suboptimal carbohydrate intake for enhanced recovery. Moreover, this review addresses the potential benefits of creatine and caffeine co-ingestion for accelerating glycogen synthesis and improving subsequent performance. Hydration strategies, including the use of milk-based beverages and electrolyte solutions, are also discussed, emphasizing their importance in maintaining fluid balance and optimizing recovery. This review also highlights the emerging role of micronutrients such as omega-3 fatty acids, antioxidants, and sodium bicarbonate in reducing muscle damage and improving acid–base balance. Evidence supports the tailored use of these nutritional strategies, particularly for athletes managing tight competition/training schedules. Future research should focus on refining individualized approaches for recovery and investigating the impact of novel supplements on subsequent performance.

**Supplementary Information:**

The online version contains supplementary material available at 10.1007/s40279-025-02213-6.

## Key Points

**Table:** 

Athletes should consume adequate carbohydrates, protein, and fluids to optimize post-exercise recovery, which is critical to maintain performance in the subsequent competition/training session when the recovery period is less than 24 h.
While creatine, caffeine, and sodium bicarbonate supplementation may improve post-exercise recovery and subsequent exercise performance in certain scenarios, their intake should be personalized to accelerate recovery for athletes preparing for the next training session or competition. The role of dietary fat and micronutrient ingestion during the post-exercise recovery period and subsequent exercise performance should be investigated in future research.
Future studies should focus on sex differences in response to carbohydrate, protein, and ergogenic aids, including creatine, caffeine, and sodium bicarbonate supplementation, during the acute recovery period in conditions such as real-world competition simulation, considering individualized approaches and training status.

## Introduction

Effective post-exercise recovery is critical for restoring physiological homeostasis, repairing muscle damage, replenishing energy stores, and facilitating adaptations that enhance athletic performance [1]. This becomes especially important when athletes are required to compete multiple times within a short period. In addition, managing fatigue and training load is essential for athletes aiming to achieve peak performance and prevent overtraining [1–3]. Nutritional strategies play a pivotal role in the recovery process by replenishing energy stores, maintaining energy availability [4], and promoting repair mechanisms and adaptive responses to exercise [5, 6]. Therefore, defining optimal nutritional interventions to promote recovery after exhaustive exercise is crucial for improving performance in subsequent bouts.

**Fig. 1:**
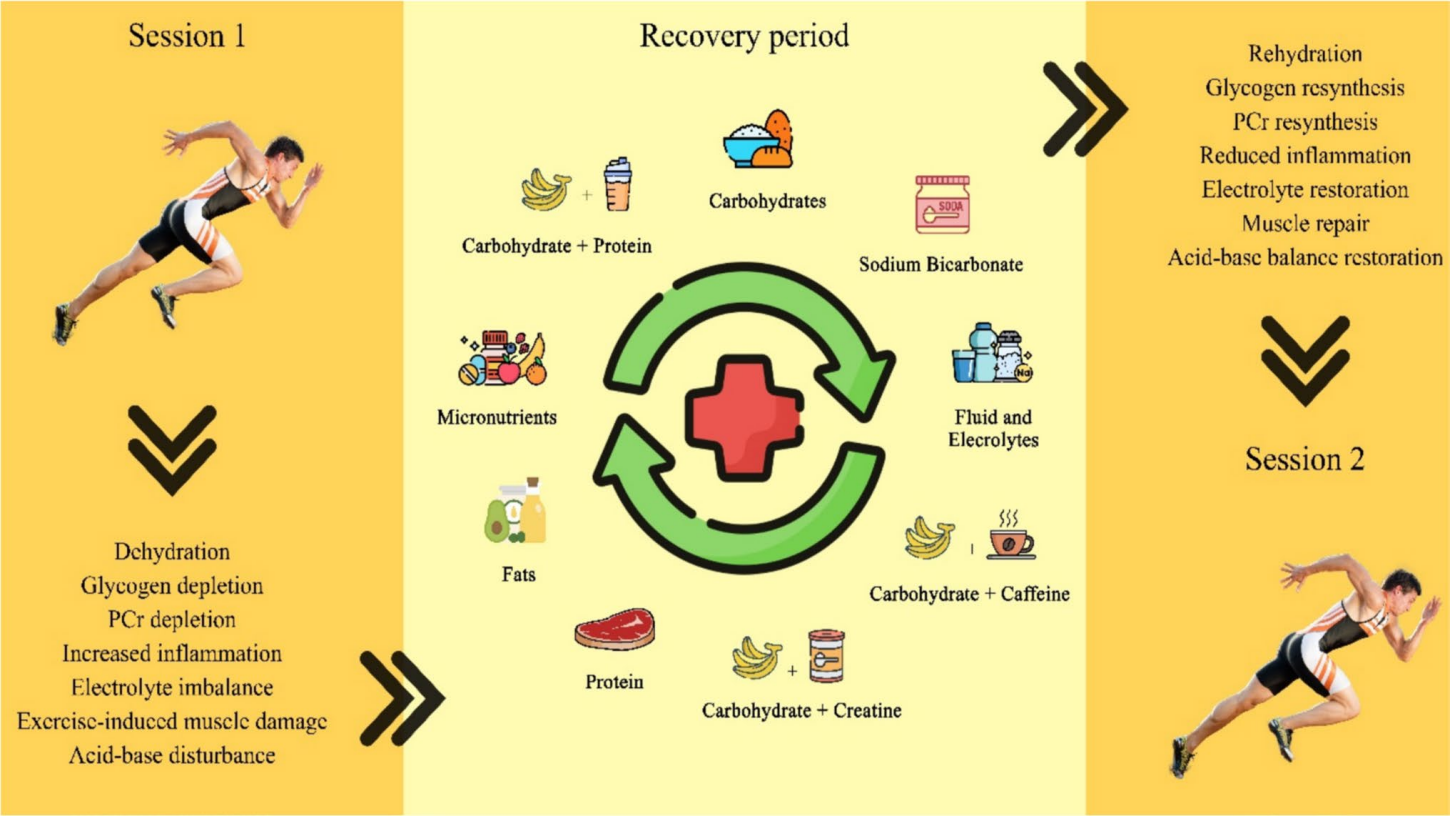
Examples of nutritional strategies to accelerate the short-term recovery period between two exercise bouts to enhance subsequent exercise sessions

Despite the growing body of literature, practical guidelines for utilizing nutrition to maximize post-exercise recovery are still evolving. The goal of this review is to synthesize the available research on nutritional strategies to enhance post-exercise recovery and subsequent exercise performance, defined as an exercise performance occurring within ~ 2–24 h of a prior bout of exhaustive exercise. Specifically, we will explore the roles of dietary macronutrients (carbohydrate, protein, and fat), micronutrients, creatine, caffeine, fluids and electrolytes, and sodium bicarbonate in accelerating recovery processes (Fig. 1). The literature search was conducted by using the following two databases: PubMed/MEDLINE and Google Scholar, using combinations of keywords and Boolean operators (“subsequent exercise performance”) AND (“carbohydrates” OR “carbohydrate and protein” OR “protein”) AND (“dietary fats” OR essential fatty acid) AND (“creatine” OR “carbohydrate and creatine” OR) AND (“sodium bicarbonate OR NaH3OC”) AND (“caffeine” OR “carbohydrate and caffeine”) AND (“micronutrients OR vitamins”). The search was performed through the titles, abstracts, and keywords of documents indexed in the databases. We classified the paper into three categories: **grade I—strong evidence**: this category includes a significant number of well-controlled trials that consistently demonstrate the effectiveness of the compound for improving post-exercise recovery; **grade II—moderate evidence**: this category contains a substantial but less consistent body of research showing the effectiveness of the compound for post-exercise recovery; and **grade III—limited evidence**: this category includes a small and/or highly ambiguous body of research that indicates the effectiveness of the compound for post-exercise recovery.

## Grade I—Strong Evidence

### Dietary Carbohydrates

#### Carbohydrate Timing and Type

**Fig. 2:**
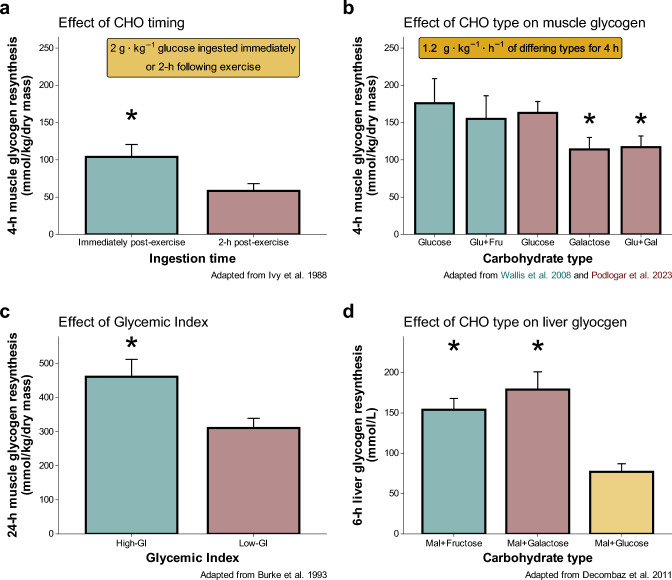
Influence of timing (**a**) and type (**b**–**d**), of carbohydrate (CHO) on glycogen resynthesis *indicates significant differenes (*p* < 0.05) from other groups within a given study. Adapted from [13, 21, 24, 27, 28]. *Fru* fructose, *Gal* galactose, *GI* glycemic index, *GLu* glucose

Replenishing glycogen stores through immediate post-exercise carbohydrate intake is crucial for recovery, especially when athletes need to be fully prepared for another training session or competition within 24 h [7–9]. The rate of restoration is influenced by both the type and timing of ingested carbohydrate (Fig. 2). Muscle glycogen demonstrates a pronounced affinity for restoration during the initial 2 h window following exercise, a process that notably occurs independent of insulin’s influence [10]. Although some muscle glycogen can be repleted in the absence of food ingestion [11], contemporary guidelines recommend ingesting 1–1.2 g·kg^−1^⋅h^−1^ of carbohydrate in the initial 4-h post-exercise window [12]. In contrast, delaying post-exercise carbohydrate ingestion by 2 h can lead to reduced muscle glycogen concentrations 4 h after exercise compared with immediate ingestion [13] and may impair exercise performance the following day [14]. Complete replenishment of muscle glycogen stores typically unfolds over a 24–36 h period post-exercise, provided that 7–12 g·kg^−1^ carbohydrate are consumed per day [15–18]. Liver glycogen resynthesis proceeds at a slower pace during the initial post-exercise hours, achieving saturation only after 11–25 h, contingent upon adherence to a high-carbohydrate diet [16].

The type of carbohydrate consumed influences the rate of muscle glycogen resynthesis following strenuous exercise. Early research demonstrated that ingestion of glucose alone increases muscle glycogen levels significantly more than fructose alone [19, 20]. However, since glucose and fructose are absorbed via distinct intestinal transporters, it was hypothesized that their combined ingestion could optimize carbohydrate absorption, minimize gastrointestinal discomfort, and potentially maximize muscle glycogen replenishment compared with glucose alone [21]. Wallis et al. [21] were the first to test this hypothesis, finding that ingesting 90 g of carbohydrate (~ 1.2 g·kg⁻^1^·h⁻^1^) in a 2:1 ratio of glucose to fructose during a 4-h post-exercise recovery phase did not enhance muscle glycogen resynthesis compared with glucose alone. This finding was confirmed by Trommelen et al. [22], who reported that co-ingesting glucose polymers with fructose or sucrose at a higher dose (1.5 g·kg^–1^⋅h^–1^) did not further augment muscle glycogen replenishment during a 5-h recovery window, despite a notable reduction in gastrointestinal complaints associated with sucrose or fructose compared with glucose alone. This finding aligns with subsequent studies comparing glucose, fructose, and sucrose in < 1 g·kg^–1^⋅h^–1^ doses during a 4–6 h post-exercise timeframe [19, 23]. More recently, Podlogar et al. [24] posited that a combination of galactose and glucose could yield similar results as compared with glucose alone in promoting muscle glycogen resynthesis. Contrary to their hypothesis, their findings revealed that glucose alone at the rate of 1.2 g·kg^−1^⋅h^–1^ facilitated a 1.3–1.6 times higher post-exercise muscle glycogen synthesis rate than galactose alone or a glucose + galactose mix. In summary, current research suggests that glucose and glucose-derived carbohydrates, with or without the addition of fructose, are the most effective sources for replenishing post-exercise muscle glycogen within a recovery window ≤ 8 h, and that addition of monosaccharide galactose results in inferior replenishment rates [24]. There is currently no evidence to recommend consuming more than 1.2 g·kg^−1^⋅h^−1^ of carbohydrates, regardless of type [22, 25]. Nevertheless, co-ingestion of glucose with fructose is preferred owing to reduced gastrointestinal complaints and the beneficial effect on liver glycogen replenishment, as discussed below [18, 22, 26] (Supplementary Material 1).

From a performance perspective, several studies have demonstrated that co-ingesting glucose and fructose-based carbohydrates can enhance the recovery of exercise capacity [29–32]. For example, Maunder et al. [33] compared the effects of 90 g of maltodextrin mixed with either glucose or fructose (in a 1.5:1 ratio) following a glycogen-depleting run. They found that the maltodextrin–fructose combination increased subsequent endurance running capacity by ~ 33% over the maltodextrin–glucose mix, highlighting the potential of glucose–fructose to enhance endurance capacity. Similarly, Gray et al. [34] reported that consuming glucose and fructose during the initial 4 h and again at 15 h of recovery from exhaustive exercise resulted in a notable increase in cycling performance capacity, ~ 27% longer compared with glucose alone. Authors suggested beneficial effects to be attributed to differences in hepatic glycogen storage on the basis of existing literature (discussed in Sect. 2.1.2), but this was not measured in these studies.

However, not all studies have shown positive outcomes with a combination of glucose and fructose. For instance, in a study examining time trial performance after a glycogen-depleting exercise, participants ingested 1.2 g·kg^−1^·h^−1^ of a glucose–fructose mix or glucose combined with maltodextrin over a 4-h recovery period before undertaking 1 h of moderate-intensity steady state cycling followed by a ~ 40-min cycling time trial [35]. Despite higher carbohydrate oxidation rates (indicating higher carbohydrate availability) following the ingestion of the glucose–fructose mixture compared with the glucose–maltodextrin mixture, performance was not improved. A mechanistic explanation for this requires further research, whereas from the applied perspective, it remains to be properly investigated if carbohydrate ingestion during subsequent exercise bout would alter the study results. In intermittent sports, Hengist et al. [36] found no improvement in either average running speed or the number of sprints after participants consumed a recovery drink containing glucose or a glucose–fructose mixture at 0.8 g·kg^−1^⋅h^−1^ plus 0.3 g·kg^−1^⋅h^−1^ of protein during 3 h of recovery after high-intensity rugby exercise test. In addition, consuming carbohydrate drinks containing glucose and fructose at doses of 30 and 60 g·h⁻^1^ did not improve subsequent taekwondo kick performance during the simulated tournament with 45 min recovery between five repeated kick tests [37]. These mixed results suggest that the type of exercise, its intensity and duration, the specific carbohydrate dosages used, and the glycogen depletion protocols might play critical roles in determining the efficacy of carbohydrate supplementation (Supplementary Material 2).

Therefore, on the basis of current research findings, it is inferred that the co-ingestion of glucose and fructose-based carbohydrates during the post-exercise recovery phase is likely more beneficial than other carbohydrate sources, given its superior efficacy in faster gastrointestinal absorption and glycogen stores and its association with reduced gastrointestinal discomfort when an optimal carbohydrate dosage of 1–1.2 g·kg^−1^⋅h^−1^ is consumed [18].

Carbohydrate types are often defined on the basis of their glycemic index (GI), a metric comparing the postprandial blood glucose response of a food to that of glucose [38]. Therefore, high-GI foods, which rapidly deliver glucose into circulation, are thought to facilitate muscle glycogen resynthesis, a process enhanced by heightened insulin activity during the post-exercise recovery phase [27]. Supporting this, research has shown that ingesting high-GI carbohydrates at a dose of 10 g·kg^–1^·24 h^–1^ significantly enhanced post-exercise muscle glycogen restoration compared with low glycemic index carbohydrates (~ 106 versus 72 mmol·kg⁻^1^ wet weight, for high- and low-GI, respectively) over a 24-h recovery period [27]. However, the influence of GI on subsequent exercise performance appears inconsistent. While one study found enhanced endurance capability following high-GI carbohydrate consumption [39], others found no effect [40–42] or even superior performance with low-GI carbohydrate intake, attributed to improved glucose stability and increased fat oxidation due to lower insulin activity [43] (Supplementary Material 1).

In summary, although high-GI carbohydrate ingestion leads to higher glycogen content after 24 h of recovery [27], during very short recovery periods the total carbohydrate dose appears more important than the GI. As discussed earlier, when fructose, which has a lower GI, is combined with glucose, it is as effective as glucose alone in promoting muscle glycogen replenishment. In addition, co-ingestion of carbohydrates with sources of fiber and fat can significantly affect the glycemic response [44]. Therefore, further research is needed to thoroughly investigate the impact of different glycemic index foods on both muscle and liver glycogen replenishment during rapid recovery time lasting 2–8 h, and on subsequent exercise performance, particularly using various glycogen-depleting exercise protocols within short recovery timeframes.

#### Liver Glycogen

Owing to methodological constraints in measuring liver glycogen levels, most sports research has focused on optimizing post-exercise muscle glycogen synthesis, despite the importance of both glycogen stores [45]. The liver plays a pivotal role in metabolic regulation during exercise by controlling blood glucose levels through glycogen storage and mobilizing non-carbohydrate sources via gluconeogenesis [26, 46]. Liver glycogen stores are depleted by approximately 40–60% during moderate to high-intensity exercise sessions that extend beyond 90 min without carbohydrate ingestion [23]. Therefore, implementing nutritional strategies that reduce liver glycogen utilization during exercise and/or promote its replenishment post-exercise may be as imperative as strategies for muscle glycogen replenishment [8, 47].

While carbohydrate ingestion during exercise does not substantially affect muscle glycogen utilization [48, 49], it can reduce or even completely suppress liver glycogen breakdown, thereby improving subsequent recovery [49, 50]. Moreover, liver glycogen resynthesis is inherently slower than that of muscle, possibly owing to its higher rate of glycogen turnover and/or distinct glycogen synthesis pathways that are contingent on hormonal fluctuations (notably insulin, glucagon, and epinephrine) [45, 47]. Research has demonstrated that the rate of liver glycogen resynthesis within the initial 2-h post-exercise period is 30–50% lower than during the subsequent 3–5 h [16]. Consequently, It has been hypothesized that certain types of carbohydrates, such as fructose and galactose, which are metabolized by the liver, might optimize the rate of liver glycogen synthesis. However, it is challenging to assess the performance implications of these changes in liver glycogen levels after consuming fructose or galactose-based carbohydrate sources by measuring subsequent exercise capacity. Supporting this hypothesis, it was observed that the addition of either fructose or galactose to maltodextrin following exercise significantly enhanced the rate of hepatic glycogen synthesis compared with a combination of maltodextrin and glucose [28] (Supplementary Material 1).

### Protein

#### Protein Type and Dose

Protein ingestion after exercise is considered one of the pillars of recovery owing to its role in promoting muscle protein synthesis (MPS), reducing muscle protein breakdown, and facilitating muscle repair and adaptation following exercise-induced damage [51–54]. Adequate protein intake is particularly essential when rapid recovery is required between training sessions or competitions [55].

The optimal dose of protein for post-exercise recovery has been extensively studied, with research suggesting that 20–40 g of high-quality protein consumed after exercise maximizes MPS rates for several hours (i.e., 3–4 h), the exact dose dependent on muscle mass involved during exercise [56–58]. While the importance of immediate post-exercise protein intake remains debated in literature [59–61], this controversy largely arises from differences in study designs and varying recovery conditions [62, 63]. Nevertheless, it is commonly recommended to rapidly increase amino acid availability following exercise to maximize MPS rates and promote muscle recovery [55, 64], with numerous studies demonstrating positive effects on recovery in endurance athletes [62, 63, 65]. Following the initial protein-based meal after exercise, it is advised that subsequent protein-rich meals follow at 3–4 h intervals [55, 66, 67]. However, it seems that more than the timing of the meals, the total amount of protein plays a more important role than the timing itself [53, 68]. Endurance athletes are recommended to consume ~ 1.2–1.8 g·kg^–1^ of high-quality protein [69–72], while those engaged in power-based sports are advised to ingest 1.7–2.2 g·kg^–1^ [73]. In addition, vegan athletes might require a higher daily intake to obtain sufficient amount of all amino acids [74]. Given the practical challenges athletes face in consuming large quantities of protein in a single meal, distributing protein intake across multiple meals throughout the day is a sensible approach. Considering the extended fasting period during sleep, a pre-sleep protein dose of ≥ 40 g has been recommended to sustain MPS rates and minimize overnight muscle protein breakdown [75, 76]. However, two studies investigating this did not observe any positive effects on recovery indices when total daily protein intake is sufficient [77, 78].

The type of protein consumed also significantly influences recovery efficacy. Amounts of essential amino acids, and especially leucine content, in protein sources seem to be the strongest predictor of MPS [79, 80]. Whey protein, a fast-digesting and leucine-rich source, has been repeatedly demonstrated to enhance MPS more effectively than casein or soy protein owing to its rapid absorption kinetics and high essential amino acid content [81]. Casein protein, by contrast, provides a slower release of amino acids and may be beneficial for sustaining protein synthesis over longer recovery periods, such as overnight [81]. Plant-based proteins such as soy, rice, and pea protein, while effective [82], generally require higher doses (25–40 g) to match the leucine threshold necessary for robust MPS stimulation owing to lower essential amino acid content [53, 74, 83].

### Carbohydrate and Protein Co-ingestion

Although carbohydrate intake is the cornerstone of glycogen resynthesis, concomitant intake of protein has been suggested to confer additional benefits, primarily owing to the insulinotropic effects of certain amino acids to reduce muscle protein breakdown [84]. However, this benefit appears to occur only when protein is added to a suboptimal amount of carbohydrate (e.g., 0.3 g·kg⁻^1^·h⁻^1^ protein and 0.9 g·kg^−1^⋅h^−1^ carbohydrate) [85]. Similarly, a meta-analysis confirmed that ingesting sufficient carbohydrate should be the priority when aiming to replenish muscle glycogen stores, reporting that co-ingestion of carbohydrate (0.86 g·kg^−1^⋅h^−^1) with protein (0.27 g·kg^−1^⋅h^−1^) did not increase muscle glycogen re-synthesis more than carbohydrate per se when ingested at 0.95 g·kg^−1^⋅h^−1^ during short-term post-exercise recovery (≤ 8 h) [86].

From a performance standpoint, co-ingesting protein with carbohydrate during recovery between two bouts of endurance exercise has shown small to moderate effects on subsequent performance compared with carbohydrate alone (e.g., improvements of ~ 1.6% in mean power output during cycling time trials and 0.6% in competitive running performance [87]. However, even a 1% improvement can be critical in elite sports, potentially making the difference between finishing fourth and securing a podium position in Olympic competitions [88]. Rustad et al. [89] reported that co-ingesting carbohydrate (0.8 g·kg⁻^1^·h⁻^1^) and protein (0.4 g·kg⁻^1^·h⁻^1^) during the first 2 h of an 18 h of recovery from strenuous cycling exercise led to ~ 14 min longer subsequent exercise capacity compared with carbohydrate intake alone (1.2 g·kg^−1^·h⁻^1^). Similarly, Dahl et al. [63] observed an ~ 8.5-min increase in exercise capacity during subsequent cycling to exhaustion at 70% peak oxygen consumption (*V*O_2peak_) when protein was co-ingested with carbohydrate within 90 min following 5 h of recovery, attributed to higher muscle glycogen synthesis rates and positive nitrogen balance compared with carbohydrate alone in trained male endurance athletes. In addition, exercise capacity during high-intensity interval cycling was significantly greater with carbohydrate-protein co-ingestion (0.8 g·kg^−1^·h⁻^1^of carbohydrates and 0.4 g·kg^−1^·h⁻^1^ of protein) compared with carbohydrate alone (1.2 g·kg^−1^·h⁻^1^) following a 2-h recovery period [90]. These positive results were mainly attributed to greater muscle glycogen synthesis or positive nitrogen balance mediated by the additional protein [85].

In contrast, recent studies have reported similar improvements in subsequent time-trial performance and high-intensity interval cycling capacity after either carbohydrates alone or carbohydrate–protein co-ingestion during 2–5 h recovery periods in both young and master endurance athletes [91, 92] (Supplementary Material 3). These discrepancies may be attributed to interindividual variations in training status and genetic factors, as well as differences in recovery durations, glycogen depletion protocols, performance tests, amounts and types of protein ingested, and methods of nutrient provision during recovery.

Taken together, combining protein with carbohydrates during recovery periods shorter than 24 h likely has a small, but positive, effect on performance, ranging from 0.6 to 1.6% [87]. Accordingly, it should be recommended as it may confer additional benefits, including positive nitrogen balance leading to increased whole-body muscle protein synthesis [93], increased protein content related to mitochondrial biogenesis [94], reduced muscle damage markers [95], and accelerated post-exercise rehydration [96]. Therefore, co-ingestion of carbohydrates and protein during post-exercise may be advantageous, especially for athletes engaging in multiple daily training sessions or with less than 24 h between bouts.

### Fluid and Electrolytes

Rehydration after exercise is another key aspect of recovery. Exercising in a dehydrated state is well-established to negatively affect both performance and health [97, 98]. During strenuous exercise in a hot or humid environment, athletes may lose more than 2 L of sweat per hour, accompanied by substantial electrolyte, extracellular, and intracellular water loss that may negatively affect processes such as the rate of post-exercise glycogen and muscle protein resynthesis [44, 99]. Therefore, re-establishing water and electrolyte balance during the post-exercise recovery period is important [100].

Rehydration is particularly important before subsequent training or competition when limited time is available between exercise bouts in environmentally challenging conditions [101, 102]. Understanding pre- and intra-exercise fluid recommendations is fundamental before addressing post-exercise hydration. Athletes are generally recommended to slowly ingest 5–7 mL·kg⁻^1^ fluids (via food and liquids) at least 4 h before exercise/competition [103]. Attempts to correct fluid loss induced by exercise should consider the sweat rate, which can vary from 0 to over 2 L·h⁻^1^, depending on factors such as environmental conditions, intensity, duration, genetics, fitness level, acclimatization to the environment, and clothing [104, 105]. Although intra-exercise hydration can mitigate fluid loss during exercise, post-exercise hydration is critical for conditions involving subsequent competitions.

If rapid recovery (< 24 h) is the aim or severe hypohydration (> 5% body mass loss) is observed, intensive intake of fluids and electrolytes should be advised to enhance recovery before subsequent competition [106]. For example, daily water and sodium losses can vary significantly and be in the range of 4–10 L and 3.5–7.0 g, respectively, in active athletes under heat stress; thus, both need to be replaced to re-establish normal total body water (i.e., achieve euhydration) [106]. Beginning subsequent exercise in a dehydrated state may impair exercise performance. Therefore, rehydration strategies between exercise bouts recommend that athletes drink fluids equivalent to 125–150% of their body mass lost during exercise [107, 108].

Maughan et al. tested different beverages using the beverage hydration index (BHI), which compares the fluid retention capacity 2 h post-ingestion compared with an equal volume of water [109], finding oral rehydration solutions, skimmed milk, whole milk, and orange juice had higher BHI values compared with water. Notably, beverages such as cola (regular and diet), tea (hot and iced), coffee, lager, sparkling water, and sports drinks did not differ from water in terms of BHI, suggesting that the volume of consumed liquid is more important than its source for hydration. Therefore, it is reasonable to use water or a mix of other beverages as post-exercise hydrational strategies, tailored to individual hydration needs and personal preferences [109].

Oral rehydration solutions and sports drinks are common strategies to improve fluid retention owing to their balanced carbohydrate and electrolyte content [110]. However, studies have observed no differences in fluid retention between post-exercise consumption of oral rehydration solutions and sports drinks following exercise-induced dehydration, even in hot conditions [111, 112] or in subsequent endurance performance [113]. Recent systematic reviews have found that dairy milk leads to a similar net fluid balance compared with carbohydrate-electrolyte sports drinks and water when consumed ad libitum and provides a higher net fluid balance when consumed in similar volumes [114, 115]. Milk-based beverages have also led to greater exercise capacity in subsequent exercise performance when compared with both noncaloric and energy-matched beverages, likely due to simultaneously increasing glycogen resynthesis and stimulating muscle protein synthesis [114]. Interestingly, nonalcoholic beer has been studied as a post-hydration drink, since it is rich in water, carbohydrates, and some minerals. However, as with other beverages, nonalcoholic beer could be considered on the basis of individual preference, but more studies are needed owing to insufficient evidence [115].

Although water is one of the most common and accessible options for post-exercise rehydration, drinking water alone may not optimize hydration status, as it has a low BHI value owing to the absence of osmotic nutrient content such as electrolytes, carbohydrate, and protein [109]. Therefore, water alone may not be the best choice to maximize subsequent exercise capacity [92, 116], especially considering that other nutrients are also essential for recovery. Conversely, factors such as an athlete’s eating behavior, thirst, palatability, and gastrointestinal comfort may influence the volume of other beverages consumed, leading to different recovery outcomes [117]. Studies examining the co-ingestion of various foods with beverages ad libitum during 2–4 h of recovery from exercise-induced dehydration have indicated that, regardless of beverage choice, similar recovery fluid balance [117, 118] and subsequent exercise performance [118] can be achieved using milk-based formulations, sports drinks, or water along with different solid meals in both males and females, despite varying dietary behaviors [117–119]. The mechanism behind this may be related to delayed gastric emptying and increased plasma osmolality, which reduces urine output and enhances fluid retention [117] (Supplementary Material 4, 5).

## Grade II—Moderate Evidence

### Sodium Bicarbonate

Sodium bicarbonate (NaHCO_3_) supplementation before exercise is widely recognized for enhancing extracellular buffering capacity when administered at doses of 0.2–0.4 g·kg⁻^1^ body mass (BM) 1–3 h prior to activity [120]. This strategy can improve high-intensity exercise performance lasting approximately 30 s to 10 min [121]. The performance enhancements observed with NaHCO_3_ supplementation are attributed to increased extracellular buffering capacity, lowered extracellular potassium (K^+^) levels, elevated sodium (Na^+^) levels before exercise, and a potential reduction in cellular oxidative stress responses [122–124]. Another mechanism that may explain the ergogenic effect of post-exercise NaHCO_3_ ingestion is its hyperhydration effect, leading to an ~ 8% increase in blood volume through increased sodium availability in the blood [125].

**Fig. 3:**
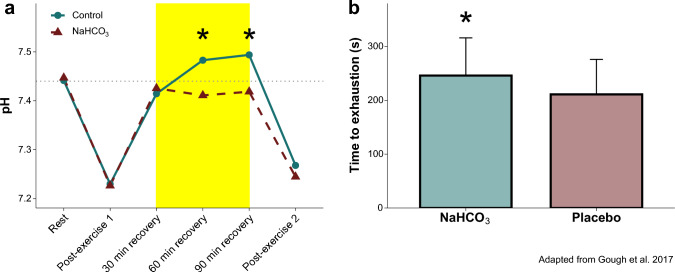
Sodium bicarbonate (NaHCO_3_) ingested 30 min following exercise to exhaustion can increase pH (**a**) and improve time to exhaustion during a subsequent exercise bout (**b**). Recovery period is highlighted in **a**. *Denotes significant differences between groups (*p* < 0.05). Adapted from Gough et al. [129]

Initial studies indicated that NaHCO_3_ ingestion before high-intensity exercise attenuated post-exercise blood acid–base imbalance during a 15-min passive recovery period compared with placebo [126] and facilitated a faster restoration of acid–base balance [127]. In addition, ingesting 0.3 g·kg⁻^1^ BM of NaHCO₃ 180 min before exercise led to ~ 3.8% improvement in subsequent performance during four repeats of 2 × 4 km cycling time trials with 40 min passive recovery conducted under hypoxic conditions as compared with placebo [128]. More recently, research has shown that administering NaHCO_3_ within approximately < 30 min following exercise can restore extracellular acid–base balance during a 75–90 min recovery period between two bouts of exercise and may improve subsequent exercise performance [129, 130] (Fig. 3), while others have reported that a shorter recovery time of 35 min may be inadequate to restored acid–base balance to improve subsequent exercise capacity [131] (Supplementary Material 6).

The lack of performance enhancement in some studies may be due to intraindividual variability in the time required to reach peak HCO₃⁻ levels and the occurrence of gastrointestinal discomfort [127, 132, 133]. De Oliveira et al. [121] established a threshold increase of 4–6 mmol/L for circulating HCO_3_⁻ levels to determine the ergogenic potential of NaHCO_3_ supplementation. This threshold depends on the time needed for circulating HCO_3_⁻ levels to rise and is influenced by factors such as the dosage and timing of ingestion [134], supplement form (i.e., solution versus capsules) [123, 135], potential adverse effects, genetic factors related to hydrogen ion (H^+^) transporters, the individual’s training status, and exercise type [136].

Taken together, administration of NaHCO_3_ after exercise can expedite the restoration of acid–base equilibrium following intense physical activity, potentially improving performance during subsequent exercise sessions if an adequate recovery period of 30–90 min is provided [124, 129, 137]. For athletes with longer recovery periods (> 90 min) who are more susceptible to severe gastrointestinal symptoms, new forms of NaHCO₃ such as enteric-coated, delayed-release capsules and mini-tablet NaHCO₃ hydrogels could be considered as they may mitigate gastrointestinal discomfort [138–140]. In addition, administering NaHCO_3_ before exercise can promote acid–base restoration and improve subsequent exercise performance, particularly when the recovery period between sessions is less than 40 min [141]. However, further research is needed to examine the effects of different NaHCO_3_ supplementation forms and their impact on HCO₃⁻ kinetics during recovery between exercise sessions. More research is needed to assess the impact of post-exercise NaHCO_3_ supplementation in brief, high-intensity exercise bouts such as combat sports.

### Creatine and Carbohydrate Co-ingestion

Creatine (α-methyl-guanidinoacetic acid) is a widely used nutritional supplement that has been extensively researched over the past two decades [142]. It is well-documented that creatine supplementation can enhance adaptations to resistance training, including increases in strength, power, and muscular endurance [143]. In addition, creatine supplementation may positively affect muscle glycogen storage in the days following glycogen-depleting exercise [144].

While the exact physiological mechanisms by which creatine augments post-exercise muscle glycogen storage are not fully understood, several plausible mechanisms have been proposed. Creatine may enhance the abundance of glucose transporter (GLUT) 4, facilitating greater glucose uptake into muscle cells [145]. It may also mitigate post-exercise inflammation, reducing markers of muscle damage and soreness [146]. This is important because exercise-induced muscle damage (EIMD) can impede muscle glycogen replenishment for up to 10 days [147], suggesting that creatine could facilitate glycogen resynthesis by alleviating EIMD. Furthermore, as an osmotically active compound, creatine can increase intracellular fluid volume [148], which may be beneficial because cell swelling has been identified as a potent stimulus for muscle glycogen synthesis [149]. However, creatine does not appear to affect muscle glycogen recovery within the initial 6 h following exercise but has been shown to increase glycogen storage over a 5-day period [144]. Other research has shown that creatine supplementation, combined with a high carbohydrate diet, led to 82% greater glycogen resynthesis within 24 h following exhaustive exercise compared with placebo [150]. Moreover, Van Loon et al. [151] observed an 18% increase in muscle glycogen content following 5 days of creatine loading (20 g·day^–1^), underscoring a notable glycogen storage boost without significant impacts on fasting plasma insulin levels or GLUT-4 mRNA/protein content. In addition, research by Nelson et al. [152] demonstrated that a moderate carbohydrate intake post-creatine loading could enhance muscle glycogen stores by 53% compared with a high-carbohydrate diet alone, further indicating the efficacy of creatine supplementation in enhancing glycogen storage, although the exact mechanism remains unclear. However, an individual’s training status may influence creatine’s efficacy regarding glycogen storage, as two studies focusing on highly trained athletes did not show significant enhancements in muscle glycogen levels following creatine supplementation [153, 154]. Specifically, trained individuals might experience reduced benefits from creatine supplementation in enhancing glycogen storage owing to their already optimized glycogen resynthesis capabilities following exercise, compared with untrained individuals [155] (Supplementary Material 7). Collectively, this evidence highlights the potential benefits and mechanisms of creatine supplementation in athletic performance and recovery, although the impact of simultaneous creatine and carbohydrate consumption on subsequent exercise performance remains debated.

In summary, the simultaneous ingestion of creatine and carbohydrate can effectively augment muscle glycogen storage, particularly after the initial 6-h post-exercise period. Despite these findings, further research is necessary to determine the impact of creatine and carbohydrate co-ingestion on endurance performance in subsequent activities, with a special focus on weight-bearing sports. It is also important to note that most studies referenced involve male subjects. Given that creatine metabolism exhibits sex-specific differences [156], there is a need for a better understanding of creatine supplementation’s effects across sex and levels of athletic training.

### Caffeine and Carbohydrate Co-ingestion

Caffeine (1,3,7-trimethylxanthine) is one of the most extensively researched performance-enhancing supplements and is known to enhance exercise performance when consumed before [157] and during prolonged exercise [158]. Recent evidence suggests caffeine could also positively affect post-exercise recovery owing to its ability to decrease muscle soreness [159] and, when paired with carbohydrates, accelerate muscle glycogen replenishment [160]. Loureiro et al. [161] proposed that caffeine might enhance muscle glycogen synthesis by activating molecular pathways related to either energy sensing or muscle contraction. Specifically, caffeine co-ingestion could lead to higher activation of adenosine monophosphate-activated protein kinase (AMPK), possibly through enhanced activation of the Ca^2+^-calmodulin-dependent protein kinase (CaMK) and the phosphorylation of acetyl-CoA carboxylase (ACC), which could collectively induce translocation of GLUT4 to the cell membrane and promote muscle glucose uptake [162, 163].

However, the evidence regarding enhanced post-exercise glycogen synthesis with caffeine co-ingestion is mixed. For example, no positive effect was observed when combining caffeine (6 mg·kg^–1^) with glucose (~ 1 g·kg^–1^⋅h^–1^) on muscle glycogen replenishment rates during a 5-h recovery period after exhaustive cycling [164]. In contrast, another study reported that muscle glycogen restoration was ~ 66% higher during 4 h of recovery with a very high dose of caffeine (8 mg·kg^–1^) and moderate carbohydrate intake (1 g·kg^–1^·h^–1^) compared with carbohydrate ingestion alone in trained endurance athletes [160]. However, both studies used carbohydrate doses below the optimal 1.2 g·kg⁻^1^·h⁻^1^ recommended for maximal glycogen resynthesis [160, 164]. Another study examined the effect of caffeine co-ingestion with a more optimal carbohydrate dose, finding no significant differences in glycogen resynthesis when 1.7 mg·kg⁻^1^·h⁻^1^ of caffeine was co-ingested with 1.2 g·kg⁻^1^·h⁻^1^ of carbohydrates (1:1 glucose and maltodextrin) during a 6-h recovery period, compared with carbohydrates alone. The authors concluded that an optimal carbohydrate dose of 1.2 g·kg⁻^1^·h⁻^1^ is sufficient to maximize glycogen replenishment rates, with no additional benefits from caffeine co-ingestion [165]. Recently, Loureiro et al. investigated the impact of caffeine co-ingestion through coffee combined with milk containing 1.2 g·kg⁻^1^ of carbohydrates and 0.3 g·kg⁻^1^ of protein on muscle glycogen resynthesis, finding that the addition of caffeine to milk resulted in approximately 153% higher rates of muscle glycogen synthesis compared with milk alone [166] (Supplementary Material 8). However, while the carbohydrate dose was considered optimal, the carbohydrate composition (milk sugars) was not, as recent evidence suggests that galactose–glucose mixtures are inferior as compared with glucose-based carbohydrates when provided at a dose of 1.2 g·kg^–1^·h^–1^ [24] (Fig. 2). Therefore, the positive effects of caffeine on muscle glycogen synthesis rates might be due to suboptimal amount or type of carbohydrate provision.

Further studies are warranted to investigate caffeine and glucose or glucose plus fructose combinations with different doses. Nevertheless, these results support the hypothesis that components within coffee could stimulate enzymes associated with muscle glycogen synthesis [161]. The positive effects of caffeine seem most apparent when muscle glycogen stores are very low and/or carbohydrate availability is suboptimal. Inconsistencies across studies may be attributed to differences in exercise protocols, glycogen depletion methods, recovery durations, dosages, and timing of carbohydrate and caffeine provision.

While caffeine–carbohydrate co-ingestion post-exercise may enhance muscle glycogen repletion, its impact on subsequent exercise performance is unclear. For instance, Taylor et al. [167] found that caffeine (6 mg·kg^–1^) combined with carbohydrate (1.2 g·kg^–1^·h^–1^ glucose) improved exercise capacity in an intermittent shuttle run test compared with carbohydrate alone (~ 48 min versus 32 min). Conversely, Andrea-Souza et al. [168] did not observe significant performance enhancements in soccer players following co-ingestion of caffeine (6 mg·kg⁻^1^) and carbohydrates (1.2 g·kg⁻^1^·h⁻^1^), possibly owing to insufficient glycogen depletion in the testing protocol. Similarly, Barzegar et al. [169] found no performance benefit with combined caffeine (6 mg·kg^–1^) and carbohydrate (0.6 g·kg^–1^) intake compared with carbohydrate alone or a placebo during a 5 × 500 m kayak test conducted 24 h after a 20-km time-trial paddle used for glycogen depletion. Moreover, caffeine ingestion alone is sufficient to enhance performance in subsequent sessions for shorter, high-intensity exercises such as wrestling [170] (Supplementary Material 9).

In summary, consuming caffeine alongside carbohydrates during the initial post-exercise recovery hours may benefit athletes who have multiple competitions in a single day or who employ training sessions with low carbohydrate availability, such as the “sleep low, train low” thought to maximize training adaptations [171]. Efficient repletion of glycogen stores after a low carbohydrate-availability exercise session is crucial for subsequent training, especially those involving high-intensity efforts [167]. It is important to note that excessive caffeine intake can lead to adverse effects, including tachycardia, anxiety, headaches, gastrointestinal issues, and a heightened risk of overnight insomnia [172, 173]. Therefore, further research focusing on glycogen storage measurements and involving larger sample sizes is necessary to clarify the impact of caffeine–carbohydrate co-ingestion on recovery and performance.

## Grade III—Limited Evidence

### Dietary Fats

Fat is primarily stored as triacylglycerol (TG) in adipose tissue, with smaller amounts inside muscle fibers (intramyocellular TG; IMTG) [174]. IMTG stores are used as an energy source mainly during early stages of endurance exercise, with the reliance on them diminishing as exercise duration extends in favor of plasma-derived substrate oxidation [175]. IMTG are also utilized as a fuel source in the post-exercise recovery window [176]. However, their availability does not seem to play a role in performance as interventions aimed at increasing IMTG have resulted in either no differences [177] or worsened [178] performance in exercise trials lasting 20–140 min. In addition, there is no to very little net utilization of IMTG during high-intensity intermittent exercise [179], which calls into question the necessity for replenishing stores prior to a competitive event. Although a low-fat diet can attenuate repletion of IMTG following exercise [180], post-exercise plasma fatty acid availability is increased independently of post-exercise dietary ingestion, mainly due to the hormonal response that stimulates adipose tissue lipolysis and skeletal muscle blood flow during the recovery period [181]. However, there is a lack of evidence to support targeted replenishment of IMTG to improve subsequent exercise performance lasting less than 3 h, but future research in the context of repeated daily ultra-endurance performance is warranted.

Ingestion of specific types of fats may have the potential to aid in the recovery of muscle function and/or influence post-exercise glycogen resynthesis. Omega-3 fatty acids, specifically eicosapentaenoic acid (EPA) and docosahexaenoic acid (DHA) are types of fats that have reported anti-inflammatory properties and the capacity for reducing EIMD and enhancing muscle function following exercise [182], providing additional rationale for their recommended use in aiding exercise recovery in a dose- and duration-dependent manner [183].

Conjugated linoleic acid (CLA) refers to a mixture of polyunsaturated fatty acids that exists as various isomers of linoleic acid (18:2), and chronic supplementation may positively influence glycogen resynthesis after exercise [184]. Dietary sources include dairy and meat from ruminant animals, which are primarily found as the *cis*-9 *trans*-11 isomer; however, synthetic isomers have also been studied [185]. While most commonly studied in the context of body composition, humans have also shown an increase in post-exercise muscle GLUT4 protein expression and post-exercise muscle glycogen resynthesis rates following 8 weeks of 3.8 g⋅day^−1^ of *trans*-10 *cis*-12 and *cis*-9 *trans*-11 CLA supplementation [184]. A study in rats supports these findings and provides some more mechanistical evidence for these findings, namely showing increased muscle glycogen synthase activity with a mixture of isomers (*cis*-9 *trans*-11, *cis*-10 *trans*-12), but not with *cis*-9 *trans*-11 isomer, suggesting physiological effects are likely isomer-specific [186]. Although consuming dietary fat in the post-exercise meal can prolong gastric emptying times [187], in the context of adequate carbohydrate ingestion, glycogen resynthesis is not influenced by the addition of fat and protein in the 3-h [188] or 24-h [189] post-exercise period. Therefore, athletes can include protein and high-quality fat in the post-exercise window without sacrificing muscle glycogen synthesis, bearing in mind that very high-fat intakes in the post-exercise window may attenuate some, but not all, signaling markers [190].

### Micronutrients

Micronutrients consumed prior to exercise, including vitamins, minerals, and phytonutrients, also have the potential to affect muscle recovery and therefore impact subsequent exercise performance. This occurs primarily via reductions in EIMD and reductions in reactive oxygen and nitrogen species (RONS) leading to improved muscle contractile function.

Several supplements may be effective in reducing EIMD, including fruit extracts such as tart cherry [191], beetroot [192], pomegranate [193], blueberry [194], watermelon [195], grape [196], and blackcurrant [197], as well as vitamin E [198], curcumin [199], cacao [200], and vitamin D [201], with the strongest evidence surrounding tart cherry, beetroot, pomegranate, and vitamin D [202, 203]. These benefits are likely due to antioxidant effects and have been observed in both trained and untrained populations [203]. However, most studies have measured indicators of muscle damage/inflammation rather than subsequent performance. Despite reductions in muscle damage and/or inflammation, improvements in performance are not always observed [191, 192].

Antioxidants have the potential to improve recovery and acute performance by reducing RONS, which impair muscle contractile force primarily through alterations in myofibrillar calcium sensitivity [204]. Often used as an umbrella term, antioxidants can exert their influence in different ways. Vitamins C and E share a common mechanism of donating a hydrogen atom to a free radical [205], whereas polyphenols stimulate stress-related cell signaling pathways and increase the expression of genes encoding proteins, such as nuclear respiratory factor 2 (NRF2), as well as pathways in skeletal muscle, leading to increased mitochondrial biogenesis [206]. Over 8000 polyphenols have been identified, including curcumin, resveratrol, quercetin, and catechins [206], and are most commonly consumed in fruits, vegetables, and spices. Although most studies of antioxidants and exercise recovery have focused on markers of oxidative stress and/or muscle damage, rather than subsequent exercise performance, quercetin supplementation for 7 days (1 g⋅day^−1^) improved time to exhaustion at 75% maximal oxygen consumption (*V*O_2max)_ by 21.4% following a 3-h recovery period after 60 min of cycling compared with a placebo and also reduced oxidative stress [207]. In contrast, 8 weeks of green tea extract supplementation (500 mg⋅day^−1^) had no effect on glycogen resynthesis, despite increases in postexercise fat oxidation and exercise-induced muscle GLUT4 protein content [208].

Free radicals produced via aerobic metabolism contribute both to exercise-induced fatigue as well as the adaptive response to exercise [209]. This means there is potential for high-dose antioxidants to blunt favorable training-induced muscle adaptations. Despite attenuations in markers of metabolic and/or mitochondrial adaptations, no impact on exercise performance outcomes has been observed in human studies when supplementing with vitamins C and/or E during controlled endurance training [210]. However, this finding could be related to small sample sizes in studies. For example, two human studies showing a blunting of some markers of training adaptations also had the largest sample sizes [211, 212], whereas a study showing no statistical effect of vitamin C on increases in *V*O_2max_ reported increases of 10.8% in the supplementation group (*n* = 5) and an increase of 22% in the group (*n* = 9) without supplementation (Cohen’s *d* =  − 1.1, [95% confidence interval (CI) − 2.2, 0.04]) [213]. Supplementation with (-)-epicatechin impaired training adaptations during 4 weeks of cycling training, as increases in *V*O_2peak_ (+ 22.6%) and succinate dehydrogenase activity (+ 59.1%) were seen in the placebo but not the supplement group, although no differences in peak power or citrate synthase activity were observed between groups [214].

Supraphysiological doses of antioxidants may be beneficial for enhancing acute recovery and subsequent performance, particularly during the competition period when adaptation is not the primary focus. However, long-term daily use should be approached with caution, as research is inconclusive regarding the extent of their effects on training adaptations, especially during the muscle adaptation training phase [210]. This could be related to the multiple pathways regulating skeletal muscle adaptations to exercise. Beyond RONS, exercise adaptations are also regulated by contraction-induced changes in mechanical strain, adenosine triphosphate (ATP) turnover, calcium flux, redox balance, and intracellular oxygen pressure [215]. As an alternative to high-dose antioxidant supplementation, whole fruits and fruit-derived juices or extracts can also provide a good source of antioxidants as well as micronutrients [216]. It is possible that interactions may occur when consuming between multiple sources of polyphenols, but practical research is challenged by a large number of active compounds in a whole-food diet and the need for longer-term studies to see meaningful effects. To optimize recovery between exercise bouts, it is recommended to consume > 1000 mg polyphenols per day either from food sources or supplements for at least 3 days [203].

## Limitations and Future Directions

Despite significant advancements in understanding nutritional strategies for post-exercise recovery, several limitations within the current body of research should be acknowledged. First, much of the existing literature is based on studies with male athletes, leading to a potential sex bias in the recommendations—a common problem within the sports science literature [217]. This disparity underscores the need for more studies involving female athletes to ensure that recovery strategies are effective across the sexes.

Another limitation is the variability in exercise protocols used across studies, which complicates the generalization of findings. For instance, research on carbohydrate replenishment often employs controlled glycogen-depleting protocols that may not reflect real-world exercise conditions when carbohydrates are also ingested during exercise or protocols are aimed at specific sports disciplines, and it is questionable how the findings translate into other disciplines. In addition, the response to various recovery strategies, such as creatine and caffeine co-ingestion, appears to be highly individualized. Factors such as training status, genetic predisposition, and diet playing key roles, suggesting a need for more personalized approaches to recovery nutrition. However, literature on personalization is currently lacking. Moreover, it is important to consider the interactions between different performance and recovery-enhancing supplements, as they often do not result in additive improvements [218]. Understanding these interactions is crucial for developing effective and safe nutritional strategies. Given that athletes such as bodybuilders and powerlifters often engage in two training sessions per day, future research is needed to explore this training strategy. Such studies should focus on individuals involved in resistance training to assess their training quality and muscle adaptation variables. Finally, a greater emphasis on the practical application of these strategies in real-world sports contexts, including team sports and ultra-endurance events, would help bridge the gap between laboratory findings and field performance. Future research should focus on translating experimental results into practical guidelines that athletes and coaches can readily implement**.**

## Conclusions

Table 1 summarises the evidence regarding the nutrition recommendations for optimizing post-exercise recovery. Table 2 provides recommendations on the basis of the duration between exercise bouts. Restoration of carbohydrate stores within the body (i.e., muscle and liver glycogen) appears to play the most important role in promoting recovery, followed by a sufficient protein intake to promote tissue regeneration and adaptive responses to exercise. While the intake of fats and micronutrients may also aid recovery, the evidence supporting their efficacy is not as strong as that for carbohydrates. Co-ingestion of caffeine with carbohydrates may accelerate post-exercise muscle glycogen resynthesis and can be beneficial for athletes competing multiple times in a single day or those engaging in training sessions with low carbohydrate availability to maximize training adaptations. However, caution is advised owing to the increased risk of sleep disturbances associated with high caffeine intake. Post-exercise creatine and carbohydrate co-ingestion may enhance muscle glycogen resynthesis but their role in improving subsequent exercise performance remains to be fully investigated. Milk-based formulations, sports drinks, oral rehydration solutions, and water, along with solid meals, may optimize post-exercise rehydration and subsequent exercise performance. Post-exercise NaHCO_3_ supplementation may enhance acid–base balance restoration and improve subsequent exercise performance, provided there is at least 30–90 min of recovery time between exercise bouts.

**Table 1:** Summary of the evidence for the effects of supplements and nutrients on recovery and subsequent exercise performance

Supplement/nutrient	Commentary
Carbohydrates	1–1.2 g·kg^–1^·h^–1^ of carbohydrate is recommended for initial 4-h post-exercise **period with glucose and fructose in a 2:1 ratio** Most of the studies used 17–20% carbohydrate solutions Small and more frequent doses are recommended
Fats	No effect on subsequent exercise performance Co-ingestion of fat and carbohydrates dose not impair glycogen resynthesis Some signaling markers may be attenuated by very high fat intake
Micronutrients	Effective in reducing exercise-induced muscle damage but no performance benefit > 1000 mg of polyphenols per day optimize recovery between exercise bouts
Protein + carbohydrate	1–1.2 g·kg^–1^·h^–1^ of carbohydrate + 20–40 g highly digestive protein advised for effective muscle glycogen resynthesis Beneficial effects may be due to higher muscle glycogen synthesis rates and/or positive nitrogen balance
Caffeine + carbohydrate	3–8 mg·kg^–1^ of caffeine + 1–1.2 g·kg^–1^·h^–1^ of carbohydrate is recommended It may reduce muscle soreness Little evidence shows that it may speed up muscle glycogen resynthesis more than carbohydrate alone
Creatine + carbohydrate	5–20 g·day^–1^ of creatine monohydrate + high carbohydrate diet may be more beneficial than carbohydrate alone for muscle glycogen resynthesis
Hydration	Replacement of 150–200% of lost sweat within 4–8 h is advised for effective rehydration Oral rehydration solutions, milk, and orange juice are better than water Cola, tea, coffee, lager, mineral water, and sports drinks are no different from water There is little evidence to suggest that glycerol improves subsequent exercise performance
Sodium bicarbonate	0.3–0.4 g·kg^–1^ of sodium bicarbonate is recommended It may improve subsequent exercise performance if recovery time is 40 min or less New forms of sodium bicarbonate, such as enteric-coated and delayed-release capsules, are recommended to reduce potential gastrointestinal discomfort

**Table 2:** Examples of post-exercise nutritional recommendations to enhance short-term recovery

Supplement/nutrient	Less than 4 h between exercise bouts	4–24 h between exercise bouts
Carbohydrate	1–1.2 g·kg^–1^·h^–1^ Small and more frequent doses are recommended	1–1.2 g·kg^–1^·h^–1^ for first 4 h, followed by an individualized approach to achieve adequate glycogen restoration
Protein	20–40 g if there is adequate time for digestion	20–40 g immediately following exercise, followed by a total daily intake of 1.2–1.8 g·kg^–1^ for endurance athletes and intake of 1.2–1.8 g·kg^–1^ for strength power athletes
Caffeine	3–8 mg·kg^–1^	3–6 mg·kg^–1^ ~ 1 h prior to the subsequent exercise bout
Sodium bicarbonate	0.3–0.4 g·kg^–1^ following the first bout of exercise	0.3–0.4 g·kg^–1^ following exercise and/or 2 h before the subsequent exercise bout
Fluid	150% body mass loss from exercise Include electrolytes if food is not also consumed	150% body mass loss from exercise Include electrolytes if food is not also consumed Can assess hydration status through urine color, frequency, and volume or by measuring urine specific gravity and osmolality

## Supplementary Information

Below is the link to the electronic supplementary material.

Supplementary file1 (PDF 173 KB)
