# Supplementary material for: Nutritional Strategies to Improve Post-exercise Recovery and Subsequent Exercise Performance: A Narrative Review
Source: Sports Med. 2025 Apr 12;55(7):1559–77. doi: 10.1007/s40279-025-02213-6 (PMC12297025; doi:10.1007/s40279-025-02213-6)
Supplement: Supplementary file 1 — Supplementary file1 (PDF 173 KB) [file 40279_2025_2213_MOESM1_ESM.pdf]

Sports Medicine Journal

Nutritional Strategies to Improve Post-Exercise Recovery and Subsequent Exercise Performance: A Narrative Review

Short title: Nutritional strategies to prepare for the next training/competition session

Alireza Naderi[1], Jeffrey A. Rothschild[2,3,] Heitor O. Santos[4], Amin Hamidvand[5], Majid S. Koozehchian[6], Abdolrahman Ghazzagh[7], Erfan Berjisian[8], Tim Podlogar[9,10]

1. Department of Sport Physiology, Faculty of Human Sciences, Borujerd Branch, Islamic Azad University, Borujerd, Iran; naderi_a@yahoo.com
2. Sports Performance Research Institute New Zealand, Auckland University of Technology, Auckland, New Zealand
3. High Performance Sport New Zealand, Auckland, New Zealand
4. Postgraduate Program, Faculdade UNIGUAÇU, Cascavel, PR, Brazil. Heitoroliveirasantos@gmail.com.
5. Department of Biological Sciences in Sport, Faculty of Sport Sciences and Health, Shahid Beheshti University, Tehran, Iran
6. Department of Kinesiology, Jacksonville State University, Jacksonville, AL 36265, USA; mkoozehchian@jsu.edu
7. Department of Sport Sciences, Faculty of Humanities, Tarbiat Modares University, Tehran, Iran
8. Department of Exercise Physiology, Faculty of Sport Sciences and Health, University of Tehran, Tehran, Iran
9. School of Sport, Exercise and Rehabilitation Sciences, College of Life and Environmental Sciences, University of Birmingham, Birmingham, United Kingdom
10. Public Health and Sport Sciences, University of Exeter, Exeter, United Kingdom

Correspondence address:
Tim Podlogar, School of Sport, Exercise and Rehabilitation Sciences, College of Life and Environmental Sciences, University of Birmingham, Birmingham, United Kingdom
Public Health and Sport Sciences, University of Exeter, Exeter, United Kingdom

Supplementary Material 1. Summary of studies exploring the effects of carbohydrate on muscle glycogen resynthesis after exhaustive exercise.

Study	Subject	VO_2max (ml.kg^{-1}.min^{-1})	Exercise Test	Treatment	Time of sampling	Result
**Blom et al. [19]**	27 physically active males	NR	Series of 20 min cycling at 75% VO_2max on bicycle ergometer and 10 min rest until exhaustion	**LG**: 0.35 g.kg^{-1} glucose in a solution with 30% concentration<br><br>**MG**: 0.70 g.kg^{-1} glucose in a solution with 30% concentration<br><br>**HG**: 1.40 g.kg^{-1} glucose in a solution with 30% concentration<br><br>**MF**: 0.70 g.kg^{-1} fructose in a solution with 30% concentration<br><br>**MS**: 0.70 g.kg^{-1} sucrose in a solution with 30% concentration	6 h after exercise	**Muscle glycogen resynthesis**: HG = MG = MS > LG = MF<br>**BGC after 1 h**: HG > MG = LG = MF = MS<br>**Insulin concentration after 1 h**: HG > MG = LG = MF = MS
**Burke et al. [27]**	5 well-trained cyclists	NR	2 h cycling at ~75% of VO_2max followed by 4 × 30 sec all-out sprints with 2 min recovery	**HGI:** 4 meals of 2.5 g.kg^{-1} CHO with high glycemic index (GI=118) during 24 h<br><br>**LGI:** 4 meals of 2.5 g.kg^{-1} CHO with high glycemic index (GI=50) during 24 h	24 h after exercise	**Muscle glycogen resynthesis:** HGI > LGI
**Van Den Bergh et al. [20]**	7 healthy active males	NR	A decreasing intermittent glycogen-depleting exercise protocol on cycle ergometer until volitional exhaustion	**FRU:** 80 g.h^{-1} fructose with 8% concentration<br><br>**GLU:** 80 g.h^{-1} glucose with 8% concentration	8 h after exercise	**Muscle glycogen resynthesis:** GLU < FRU
**Burke et al. [194]**	8 well-trained male triathletes	NR	2 h cycling at ~75% of VO_2max followed by 4 × 30 sec all-out sprints with 2 min recovery	**Gorging diet:** 4 large meals consist 2.5 g.kg^{-1} of CHO during 24 h<br><br>**Nibbling diet:** total of 10 g.kg^{-1} of CHO during 24 h in 16 meals	24 h after exercise	**Muscle glycogen resynthesis:** Gorging diet = Nibbling diet

**Ivy et al. [13]**	12 healthy active males	NR	70 min cycling at ~68% of VO_2max followed by 6 × 2 min intervals at 88% VO_2max with 2 min recovery	**P-EX:** 2 $g.kg^{-1}$ of glucose immediately after exercise<br><br>**2P-EX**: 2 $g.kg^{-1}$ of glucose 2 h after exercise	2 and 4 h after exercise	**Muscle glycogen resynthesis:** P-EX > 2P-EX
**Wallis et al. [21]**	6 healthy endurance-trained males	67.0 (±3.0)	A decreasing intermittent glycogen-depleting exercise protocol on cycle ergometer until volitional exhaustion	**GLU+FRU:** 0.8 $g.kg^{-1}.h^{-1}$ of glucose and 0.4 $g.kg^{-1}.h^{-1}$ of fructose solution with 18% concentration<br><br>**GLU:** 1.2 $g.kg^{-1}.h^{-1}$ of glucose solution with 18% concentration	1 and 4 h after exercise	**Muscle glycogen resynthesis:** GLU > GLU+FRU<br>**Muscle glycogen synthesis rates:** GLU = GLU+FRU<br>**BGC:** GLU = GLU+FRU<br>**Insulin:** GLU = GLU+FRU<br>**[BLa^-]:** GLU < GLU+FRU
**Decombaz et al. [28]**	10 well-trained male cyclists	NR	A decreasing intermittent glycogen-depleting exercise protocol on cycle ergometer until volitional exhaustion	**MAL+FRU:** 69 $g.h^{-1}$ maltodextrin and fructose with 2:1 ratio and 15% concentration<br><br>**MAL+GAL**: 69 $g.h^{-1}$ maltodextrin and galactose with 2:1 ratio and 15% concentration<br><br>**MAL+GLU**: 69 $g.h^{-1}$ maltodextrin and glucose with 2:1 ratio and 15% concentration	6.5 h after exercise	**Liver glycogen resynthesis:** MAL+FRU = MAL+GAL > MAL+GLU
**Trommelen et al. [22]**	14 well-trained male cyclists	NR	A decreasing intermittent glycogen-depleting exercise protocol on cycle ergometer until volitional exhaustion	**GLU+FRU**: 0.6 $g.kg^{-1}.h^{-1}$ glucose + 0.6 $g.kg^{-1}.h^{-1}$ maltodextrin + 0.3 $g.kg^{-1}.h^{-1}$ fructose<br><br>**GLU+SUC**: 0.6 $g.kg^{-1}.h^{-1}$ glucose + 0.6 $g.kg^{-1}.h^{-1}$ maltodextrin + 0.3 $g.kg^{-1}.h^{-1}$ sucrose<br><br>**GLU**: 0.9 $g.kg^{-1}.h^{-1}$ glucose + 0.6 $g.kg^{-1}.h^{-1}$ maltodextrin	5 h after exercise	**BGC**: GLU+FRU = GLU+SUC = GLU<br>**Insulin**: GLU+FRU = GLU+SUC > GLU<br>**[BLa^-]**: GLU+FRU = GLU+SUC > GLU<br>**Muscle glycogen resynthesis**: GLU+FRU = GLU+SUC = GLU<br>**GI score**: GLU+FRU = GLU+SUC < GLU
**Fuchs et al. [25]**	15 well-trained male cyclists	61.5 (±5.2)	A decreasing intermittent glycogen-depleting exercise protocol on cycle ergometer until volitional exhaustion	**SUC**: 1.5 $g.kg^{-1}.h^{-1}$ sucrose solution with ~20% concentration<br><br>**GLU**: 1.5 $g.kg^{-1}.h^{-1}$ glucose (0.9 $g.kg^{-1}.h^{-1}$ dextrose monohydrate and 0.6 $g.kg^{-1}.h^{-1}$ maltodextrin) solution with ~20% concentration	5 h	**Liver glycogen resynthesis**: SUC = GLU<br>**Muscle glycogen resynthesis**: SUC = GLU<br>**BGC** **at 65, 75 and 90 min**: SUC < GLU<br>**Insulin** **at 45, 75 and 90 min**: SUC < GLU<br>**NEFA**: SUC = GLU

**Podlogar et al. [195]**	9 healthy endurance-trained participants (male = 7, female = 2)	51.1 (±8.7)	A decreasing intermittent glycogen-depleting exercise protocol on cycle ergometer until volitional exhaustion	**GLU+GAL**: 0.8 $g.kg^{-1}.h^{-1}$ glucose and 0.4 $g.kg^{-1}.h^{-1}$ galactose<br><br>**GLU**: 1.2 $g.kg^{-1}.h^{-1}$ glucose<br><br>**GAL**: 1.2 $g.kg^{-1}.h^{-1}$ galactose	4 h	**Muscle glycogen resynthesis**: GLU > GLU+GAL = GAL<br>**BGC**: GLU+GAL = GAL > GLU<br>**Insulin**: GLU+GAL = GLU > GAL = ↔<br>**$[BLa^-]$**: GLU+GAL = GLU > GAL<br>**Phosphorylation of**:<br>• **Akt ^{pThr308}**: GLU+GAL = GLU > GAL<br>• **Akt^{pSer473}**: GLU+GAL = GLU = GAL<br>• **GS^{pSer641}**: GLU+GAL = GLU = GAL<br>• **GSK^{PSer21}**: GLU+GAL = GLU = GAL

Abbreviations: Akt^{pThr308}: Phosphorylated Akt at threonine 308; Akt^{pSer473}: Phosphorylated Akt at serine 473; BGC: Blood glucose concentration; [BLa-]: Blood lactate concentration; CHO: Carbohydrate; FRU: Fructose; GLA: Galactose; GLU: Glucose; GSK^{PSer21}: Phosphorylated Glycogen synthase kinase-3 at serine 21; HGI: High glycemic index; LG: Large glucose solution; LGI: Low glycemic index; MAL: Maltodextrin; MF: Medium fructose solution; MG: Medium glucose solution; MS: Medium sucrose solution; NEFA: Non-esterified fatty acid concentrations; NR: Not reported; P-EX: Post exercise; SUC: Sucrose; 2P-EX: Two hours post exercise.

Supplementary Material 2. Summary of studies exploring the effects of carbohydrate on subsequent exercise performance after exhaustive exercise.

Study	Subject	VO_2max (ml.min^{-1}.kg^{-1})	Pre-conditioning	Recovery Period	Treatment	Subsequent Exercise	Main findings
**Casey et al. [23]**	10 healthy males	NR	TTE test on bicycle ergometer at 70% of VO_2max	4 h	**GLU:** 1 g.kg^{-1} glucose solution with 18.5% concentration<br><br>**SUC:** 1 g.kg^{-1} sucrose solution with 18.5% concentration<br><br>**PLA:** non-caloric flavored water	Cycling TTE test at 70% of VO_2max	**TTE:** GLU = SUC = PLA<br>**Liver glycogen resynthesis:** GLU = SUC > PLA<br>**Muscle glycogen resynthesis:** GLU = SUC = PLA<br>**CHO oxidation**: GLU = SUC > PLA<br>**BGC**: GLU = SUC > PLA<br>**Epinephrine**: GLU = SUC = PLA<br>**Norepinephrine**: GLU = SUC = PLA<br>[BLa-]: GLU = SUC = PLA<br>**Ammonia concentration**: GLU = SUC = PLA<br>**RER**: GLU = SUC > PLA
**Erith et al. [40]**	7 male semi-professional soccer players	58.0 (±1.0)	LIST	22 h	**HGI-diet:** 8 g.kg^{-1}.d^{-1} CHO with GI = 70<br><br>**LGI-diet:** 8 g.kg^{-1}.d^{-1} CHO with GI = 35	LIST	**TTE_{LIST}**: HGI-diet > LGI-diet<br>**Sprint performance**: HGI-diet = LGI-diet<br>**Distance covered**: HGI-diet = LGI-diet
**Wong et al. [39]**	7 male trained endurance runners	62.1 (±2.2)	TTE test on level treadmill at workload corresponding to 70% VO_2max	4 h	**HGI**: 1.5 g.kg^{-1} of CHO with GI = 77<br><br>**LGI**: 1.5 g.kg^{-1} of CHO with GI = 37	TTE test on level treadmill at workload corresponding to 70% VO_2max	**TTE**: HGI > LGI<br>**[BLa$^-$]**: HGI = LGI<br>**HR**: HGI = LGI<br>**RPE**: HGI = LGI<br>**RER**: HGI = LGI<br>**CHO oxidation**: HGI = LGI<br>**Fat oxidation**: HGI = LGI
**Brown et al. [196]**	7 male amateur cyclists	NR	A decreasing intermittent glycogen-depleting exercise protocol on cycle ergometer until	3 h	**LGI**: a low glycemic index meal with (672kcal ,140g CHO ,13 g fat ,27 g PRO, GI=40)	5-km cycling TT on cycle ergometer	**TT**: HGI = LGI<br>**Insulin**: HGI > LGI<br>**RER**: HGI > LGI<br>**CHO oxidation**: HGI > LGI<br>**Fat oxidation**: HGI < LGI

			volitional exhaustion		**HGI**: a high glycemic index meal with (774 kcal, 140 g CHO,12 g fat, 31 g PRO, GI=72)		
**Alghannam et al. [197]**	10 recreationally endurance trained individuals (male = 9, female = 1)	61.00 (±1.0)	TTE test on treadmill at workload corresponding to 70% of VO_2max	4 h	**H-CHO:** 1.2 $g.kg^{-1}.h^{-1}$ CHO solution with 12% concentration<br><br>**L-CHO:** 0.3 $g.kg^{-1}.h^{-1}$ CHO solution with 3% concentration	TTE test on treadmill at workload corresponding to 70% of VO_2max	**TTE:** H-CHO > L-CHO **Muscle glycogen resynthesis:** H-CHO > L-CHO **Insulin:** H-CHO > L-CHO **[BLa^-]:** H-CHO = L-CHO **CHO oxidation:** H-CHO > L-CHO **Fat oxidation:** H-CHO < L-CHO
**Maunder et al. [33]**	8 trained male endurance runners and triathletes	NR	TTE test on treadmill at workload corresponding to 70% of VO_2max	4 h	**GLU+MAL:** 300 ml solution with 18 g glucose and 27 g maltodextrin<br><br>**FRU+MAL:** 300-ml solution with 18 g fructose and 27 g maltodextrin	TTE test on treadmill at workload corresponding to 70% of VO_2max	**TTE:** FRU+MAL < GLU-MAL **CHO oxidation:** FRU+MAL < GLU-MAL **BGC:** FRU+MAL = GLU-MAL **[BLa^-]:** FRU+MAL < GLU-MAL
**Podlogar et al. [198]**	11 endurance-trained participants (Male = 8, Female = 3)	56.9 (±4.8)	A decreasing intermittent glycogen-depleting exercise protocol on cycle ergometer until volitional exhaustion	4 h	**GLU+MAL:** 1.2 $g.kg^{-1}.h^{-1}$ of 1:1.5 dextrose: maltodextrin ratio<br><br>**FRU+MAL:** 1.2 $g.kg^{-1}.h^{-1}$ of 1:1.5 fructose: maltodextrin ratio	60 min steady state cycling at 50% Wmax, followed by TT to total amount work of 2,400 J	**TT:** GLU+MD = FRU+MD **CHO oxidation:** GLU+MD < FRU+MD
**McCarthy et al. [199]**	10 recreationally-active males	47.0 (±5.4)	5 × 4-min intervals at ~80% VO_2peak separated by 2-min at ~40% VO_2peak and	2 h	**CHO:** 1.2 $g.kg^{-1}.h^{-1}$ of CHO (sucrose, dextrose, maltodextrin, modified corn starch)	5 × 4-min intervals at ~80% VO_2peak separated by 2-min at ~40% VO_2peak	**TTE:** CHO > PLA

			ended with an TTE test at ~90% VO_2peak		**PLA:** low-calorie drink	and ended with an TTE test at ~90% VO_2peak	
**Gray et al. [34]**	8 trained male cyclists (male = 5, female = 3)	Short recovery time experiment: 66.9 (±6.1) Overnight recovery experiment: 62.9 (±9.8)	A decreasing intermittent glycogen-depleting exercise protocol on cycle ergometer until volitional exhaustion	Short recovery time experiment: 4 h Overnight recovery experiment: 15 h	**GLU+FRU:** 0.6 $g.kg^{-1}.h^{-1}$ glucose + 0.6 $g.kg^{-1}.h^{-1}$ fructose solution with 20% concentration **GLU:** 1.5 $g.kg^{-1}.h^{-1}$ glucose solution with 20% concentration	Short recovery time experiment: TTE test at 70% Wmax Overnight recovery experiment: TTE test at 65% Wmax	**Short recovery time experiment:** • **TTE:** GLU+FRU > GLU • **CHO oxidation during rest:** GLU+FRU > GLU • **CHO oxidation during TTE:** GLU+FRU = GLU • **GI score:** GLU+FRU = GLU • **[BLa$^-$]:** GLU+FRU = GLU • **BGC:** GLU+FRU = GLU **Overnight recovery experiment:** • **TTE:** GLU+FRU > GLU • **[BLa$^-$]:** GLU+FRU = GLU • **BGC:** GLU+FRU = GLU • **CHO oxidation:** GLU+FRU = GLU Fat oxidation: GLU+FRU = GLU
**Hengist et al. [36]**	12 male Rugby Union players	NR	8 SSG with high intensity	3 h	**GLU+FRU:** 0.4 $g.kg^{-1}.h^{-1}$ fructose, 0.4 $g.kg^{-1}.h^{-1}$ glucose solution with 20% concentration + 0.3 $g.kg^{-1}.h^{-1}$ PRO	8 SSG with high intensity	**Mean speed during sessions:** GLU+FRU = GLU **GI score:** GLU+FRU = GLU

					**GLU:** 0.8 g.kg^{-1}.h^{-1} glucose solution with 20% concentration + 0.3 g.kg^{-1}.h^{-1} PRO		
**Naderi et al. [37]**	11 junior male professional taekwondo athletes	NR	5 times simulated taekwondo effort test (STET) with 45-min rest, STET included 3 rounds that lasting 200 sec with 1-min rest between rounds.		**Immediately after each STET:**<br><br>**C45:** 45 g of 2:1 glucose: fructose solution (60 g.h^{-1}) with 7.5% Concentration<br><br>**C22.5:** 22.5 g of 2:1 glucose: fructose solution (30 g.h^{-1}) with 3.5% Concentration<br><br>**PLA:** beverage contained 2.2 g No caloric sweetener powder		**Total kicks:** C45 = C22.5 = PLA<br>**Total successful kicks:** C45 = C22.5 = PLA<br>**Successful kick %:** C45 = C22.5 = PLA<br>**RPE:** C45 = C22.5 = PLA<br>**BGC:** C45 = C22.5 > PLA
**Podlogar et al. [200]**	8 trained male cyclists	62.2 (±5.4)	2 × 2-min at RCP, followed by 4 × 8-min at 104% RCP with 4 min of active recovery (i.e., easy cycling). After the successful completion participants cycled for 60 min at workload corresponding to 90% of VT1.	> 12 h	**2 h before second exercise:**<br><br>**GLU+RICE:** 2 g.kg^{-1} glucose and rice, in 1:2 ratio<br><br>**FRU+RICE:** 2 g.kg^{-1} fructose and rice, in 1:2 ratio	TTE test at VT1	**TTE:** FRU+RICE > GLU+RICE<br>**BGC:** FRU+RICE = GLU+RICE<br>**[BLa$^-$]:** FRU+RICE = GLU+RICE<br>**CHO oxidation:** FRU+RICE > GLU+RICE

Abbreviations: BGC: Blood glucose concentration; CHO: Carbohydrate; GI: Glycemic index; GI score: Gastrointestinal discomfort scores; LIST: Loughborough Intermittent Shuttle Test; NR: Not reported; PRO: Protein; RCP: Respiratory compensation point; RPE: Rate of perceived

exertion; SSG: Small sided games; TT: Time trial; STET: Simulated Taekwondo effort test; C45: Solution containing 45 g of carbohydrate; C22.5: Solution containing 22.5 g of carbohydrate; PLA: Placebo; VO_2peak: Peak oxygen uptake; H-CHO: High carbohydrate solution; L-CHO: Low carbohydrate solution; MAL: Maltodextrin; VO_2max: Maximal oxygen uptake; HR: Heart rate; VT1: First ventilatory threshold; Wmax: Peak power achieved during incremental cycling test; [BLa-]: Blood lactate concentration during or after exercise; TTE: Time to exhaustion; TTE_{LIST}: Time to exhaustion in Loughborough Intermittent Shuttle Test; FRU: Fructose; GLA: Galactose; GLU: Glucose; HGI: High glycemic index; LGI: Low glycemic index; NR: Not reported; RER: Respiratory exchange ratio; SUC: Sucrose.

Supplementary Material 3. Summary of studies exploring the effects of carbohydrate and protein co-ingestion on subsequent exercise performance after exhaustive exercise.

Study	Subject	VO_2max (ml.kg^{-1}.min^{-1})	Pre-conditioning	Recovery Period	Treatment	Subsequent Exercise	Result
**Rustad et al. [56]**	8 endurance-trained males	NR	**Warm up:** 3 × 4 min cycling at 50, 55 and 60% of VO_2max. **Interval Main exercise:** 20-min intervals at workload corresponding to ~72% of VO_2max with 5-min rest between intervals. After voluntary exhaustion, subjects were given 5-min of rest before completing a maximal number of 1 min intervals at 90% of VO_2max with 1 min rest between intervals.	18 h	**CHO:** 0.6 g.kg^{-1}.h^{-1} maltodextrin + 0.6 g.kg^{-1}.h^{-1} glucose solution with 17% concentration<br><br>**CHO+PRO:** 0.4 g.kg^{-1}.h^{-1} maltodextrin + 0.4 g.kg^{-1}.h^{-1} glucose solution with 7% concentration + 0.4 g.kg^{-1}.h^{-1} of PRO whey<br><br>**PLA:** non-caloric flavored water	**Warm up:** 3 × 4 min at 50, 55 and 60% of VO_2max. **Test:** TTE test at 72% of VO_2max	**TTE:** CHO+PRO > CHO > PLA<br>**RPE:** CHO+PRO = CHO = PLA<br>**RER:** CHO+PRO = CHO > PLA<br>**CHO oxidation:** CHO+PRO > CHO > PLA<br>**BGC_{15min}:** CHO+PRO = CHO > PLA<br>**[BLa^-]:** CHO+PRO = CHO = PLA<br>**Insulin:** CHO+PRO = CHO = PLA<br>**FFA:** CHO+PRO = PLA < CHO<br>**Muscle damage biomarkers:**<br>• **CK** ↔<br>• **LDH** ↔<br>• **Myoglobin** ↔<br>**Nitrogen balance:** CHO+PRO > CHO > PLA<br>**Valine concentration:** CHO+PRO > CHO
**Dahl et al. [57]**	9 endurance trained males	58.1 (±1.7)	TTE test on bicycle ergometer at 70% of VO_2peak	1.5 h	**CHO:** 0.6 g.kg^{-1}.h^{-1} maltodextrin + 0.6 g.kg^{-1}.h^{-1} glucose solution with 17% concentration<br><br>**CHO+PRO:** 0.4 g.kg^{-1}.h^{-1} maltodextrin + 0.4 g.kg^{-1}.h^{-1} glucose solution with ~7% concentration + 0.4 g.kg^{-1}.h^{-1} of PRO whey	TTE test on bicycle ergometer at 70% of VO_2peak	**TTE:** CHO+PRO > CHO<br>**Nitrogen balance:** CHO+PRO > CHO = 0<br>**Phosphorylation of:**<br>• **Akt Ser^{473}** ↔<br>• **p70s6k Thr^{389}** ↔<br>• **TSC2 Thr^{1462}** ↔<br>• **TBC1D4/AS160 Ser^{588}** ↔

**Goldstein et al. [58]**	25 recreationally active males	43.8 (± 6.9)	5 × 4-min at 70-80% of PPO with 2 min of active recovery at 50W, followed by TTE test at 90% PPO	2 h	**CHO:** 1.2 g.kg^{-1}.h^{-1} CHO solution<br><br>**CHO+PRO:** 0.8 g.kg^{-1}.h^{-1} CHO solution + 0.4 g.kg^{-1}.h^{-1} of PRO whey<br><br>**PLA:** non-caloric flavored water	5 × 4-min at 70-80% of PPO with 2 min of active recovery at 50W, followed by TTE test at 90% PPO	**TTE:** CHO+PRO > CHO > PLA<br>**RER:** CHO > CHO+PR = PLA
**Goldstein et al. [201]**	22 male masters class endurance athletes	48.6 (±6.7)	5 × 4-min at 70-80% of PPO with 2 min of active recovery at 50W, followed by TTE test at 90% PPO	2 h	**CHO:** 1.2 g.kg^{-1}.h^{-1} CHO solution<br><br>**CHO+PRO:** 0.8 g.kg^{-1}.h^{-1} CHO solution + 0.4 g.kg^{-1}.h^{-1} of PRO whey<br><br>**PLA:** non-caloric flavored water	5 × 4-min at 70-80% of PPO with 2 min of active recovery at 50W, followed by TTE test at 90% PPO	**TTE:** CHO+PRO = CHO > PLA<br>**HRRi:** CHO+PRO = CHO > PLA

Abbreviations: TTE: Time to exhaustion; RPE: Rate of perceived exertion; RER: Respiratory exchange ratio; FFA: Free fatty acids; BGC_{15min}: Blood glucose concentration at minute 15; CHO: Carbohydrate; PRO: Protein; Akt^{Ser473}: Akt phosphorylated at serine 473; $p70s6k^{Thr389}$: Phosphorylated p70S6 kinase at threonine 389; $TSC2^{Thr1462}$: Tuberous sclerosis complex 2 phosphorylated at threonine 1462; $TBC1D4/AS160^{Ser588}$: TBC1 domain family member 4 (also known as AS160) phosphorylated at serine 588; LDH: Lactate dehydrogenase; CK: Creatine kinase; PPO: Peak power output; BGC: Blood glucose concentration; HRRi: Heart rate recovery index, accounts for heart rate recovery relative to work; LDH: Lactate dehydrogenase; NR: Not reported; PRO: Protein; RPE: Rate of perceived exertion; [BLa-]: Blood lactate concentration after exercise; ↔: No difference.

Supplementary Material 4. Summary of studies exploring the effects of fluid and electrolytes on post post-exercise rehydration.

Study	Subject	VO_2max (ml.kg^{-1}.min^{-1})	Exercise Test	Treatment	Time of sampling	Main Findings
**Maughan et al. [87]**	8 healthy active volunteers (male = 5, female = 3)	46.5 (±3.1)	Series of 10 min cycling at ~60% VO_2max in 34°C, 55% RH; 5-min rests between sessions until ~2% body mass loss through dehydration.	**ORS**: Consumed equal to 150% of sweat loss volume via a sports drink containing 6.4 g.L^{-1} CHO, 21 mmol.L^{-1} sodium, and 3.4 mmol.L^{-1} potassium.<br><br>**MEAL+ORS**: Consumed equal to 150% of sweat loss volume via a sports drink containing 6.4 g.L^{-1} CHO, 21 mmol.L^{-1} sodium, and 3.4 mmol.L^{-1} potassium + a meal of chili beef and rice, providing 63 kJ.kg^{-1} body mass (53% carbs, 28% fat, 19% PRO).	Blood and urine samples were collected at the end of the rehydration period and at 1, 2, 4, and 6 h post rehydration.	**Serum Osmolality**: ORS > MEAL+ORS<br>**Cumulative Urine Output**: MEAL+ORS < ORS<br>**Urine Production (2-hour period)**: MEAL+ORS < ORS<br>**Urinary Na+ and K+ Excretion**: MEAL+ORS > ORS<br>**Water Retention**: MEAL+ORS > ORS
**Maughan et al. [77]**	72 healthy active males	NR	-	1L of still water, ORS, full-fat milk, skimmed milk, cola, tea, coffee, lager, orange juice, sparkling water, and sports drink ingested over 30 minutes.	Urine output collected over the following 4 h after ingestion	**Cumulative Urine Output**: ORS < Full-fat milk < Skimmed milk < Still water = Cola = Tea = Coffee = Lager = Orange Juice = Sparkling Water = Sports Drink<br>**BHI after 2 h**: Skimmed milk > ORS > Full-fat milk
**Schleh et al. [80]**	10 aerobically fit males	53.9 (±5.9)	45 min of walking at 50% VO_2max	**ORS**: Consumed equal to 150%	Urine output collected	**Plasma volume change**: ORS = SD<br>**BGC**: SD > ORS

			at (39ºC, 30% RH) + 30 min rest period in the heat wearing firefighter personal protective clothing + 45 min of walking at 50% VO_2max at (39ºC, 30% RH) + 30 min rest period in the heat wearing firefighter personal protective clothing	of sweat loss volume via an ORS (60.9 mmol.L^{-1} sodium and 34 g.L-1 CHO)<br><br>**SD**: Consumed equal to 150% of sweat loss volume via an ORS (18.4 mmol.L^{-1} sodium and 59 g.L-1 CHO)<br><br>**W**: Consumed equal to 150% of sweat loss volume via a flavored water	over the following 90 min after trial	**Fat oxidation post-exercise**: ORS > SD<br>**Sweat rate**: ORS = SD<br>**Dehydration**: ORS = SD
**Ly et al. [79]**	26 physically active males	56.4 (± 6.9)	90 min indoor intermittent exercise split into three 25 min sessions on treadmill, bike, and elliptical after a 2 min warm-up. Each session included walking (~3 mph), jogging (~7 mph), and running (~10 mph) or equivalent intensities on the bike/elliptical.	**ORS**: Consumed equal to 100% of sweat loss volume via an ORS (45 mmol.L^{-1} sodium and 25 g.L-1 CHO)<br><br>**SD**: Consumed equal to 100% of sweat loss volume via an ORS (18 mmol.L^{-1} sodium and 60 g.L-1 CHO)<br><br>**W**: Consumed equal to 100% of sweat loss volume via a flavored water	Urine output collected over the following 3.5 h after ingestion	**Fluid Retention**: ORS = SD > W<br>**Urine Production in first 1 h**: ORS < W = SD<br>**Fluid Retention at 3.5 h**: ORS ≥ SD > W
**Campagnolo et al. [202]**	10 endurance trained males	63.0 (±7.2)	5 min warm-up cycling at 100 W followed by 20 min at 65% PPO. If ~1.8% body weight loss was not achieved, workload reduced by	**MEAL+SD**: Self-serve food items included sports bars, fresh fruit, breads and condiments ingestion + 73 g.L^{-1} CHO +	4 h	**Fluid retention**: MEAL+SD = MEAL+S = MEAL+W<br>**Total energy intake**: MEAL+S = MEAL+SD > MEAL+W<br>**Total fluid intake**: MEAL+SD > MEAL+W > MEAL+S

		~5% every 10 min to a tolerable minimum of 50% PPO. Exercise performed at 23°C, 70% RH.	280 mg.L^{-1} sodium<br><br>**MEAL+S**: Self-serve food items included sports bars, fresh fruit, breads and condiments ingestion + Sustagen a milk-based liquid meal supplement containing 176 g.L^{-1} CHO + 65 g.L^{-1} PRO + 2 g.L^{-1} fat + 670 mg.L^{-1} sodium<br><br>**MEAL+W**: Self-serve food items included sports bars, fresh fruit, breads and condiments ingestion + water		**Total urine output**: MEAL+S < W < MEAL+SD<br>**Bloating perception**: MEAL+S > MEAL+SD > W<br>**Thirst perception**: MEAL+SD < MEAL+S

Abbreviations: BGC: Blood glucose concentration; BHI: Beverage hydration index; CHO: Carbohydrate; MEAL+S: Meal with Sustagen; NR: Not reported; ORS: Oral rehydration solution; PPO: Peak power output; RH: Relative humidity; SD: Sports drink; TTE: Time to exhaustion; TT: Time trial; VO_2max: Maximum oxygen uptake; W: Water

Supplementary Material 5. Summary of studies exploring the effects of fluid and electrolytes on subsequent exercise after primary exercise.

Study	Subject	VO_2max ($ml.min^{-1}.kg^{-1}$)	Pre-conditioning	Recovery Period	Treatment	Subsequent Exercise	Main findings
**McCartney et al. [86]**	16 trained cyclists (male = 8, female = 8)	Male: 54±6 Female: 50±7	1 h cycling on ergometer bike: 6 × 10 min (8 min at 50 to 55% of PPO, 1 min at 70% of PPO, and 1 min active recovery)	4 h	**W**: Self-serve CHO ingestion + Water that containing 3 $mg \cdot L^{-1}$ sodium<br><br>**SD**: Self-serve CHO ingestion + Sport drink (58 $g.L^{-1}$ CHO + 3 $mg.L^{-1}$ sodium)	45 min cycling at 50 to 55% of PPO, followed by an incremental test. The incremental test consisted of a 1 W increase every 6 s until volitional exhaustion	**Fluid balance**: W = SD **Energy intake from self-serve food**: W < SD **TTE**: W = SD
**Fan et al. [81]**	9 physically active males	55.0 (± 6.0)	75 min cycling at 65% VO_2peak (temperature: 30.4 ±0.3º C, RH: 76 ±1%, simulated wind speed: 8.0 ±0.6 $m.s^{-1}$)	3 h	**SD**: Consumed equal to 150% of sweat loss volume via a sports drink solution containing 62 $g.L^{-1}$ CHO + 31 $mmol.L^{-1}$ sodium<br><br>**ORS**: Consumed equal to 150% of sweat loss volume via an oral rehydration solution containing 33 $g.L^{-1}$ CHO + 60 $mmol.L^{-1}$ sodium<br><br>**W**: Consumed equal to 150% of sweat loss volume via water	45 min cycling at a 65% VO_2peak followed by a 20 km TT test	**TT**: W = SD = ORS **Fluid Retention**: ORS > SD > W **Palatability**: W = SD = ORS

Abbreviations: BGC: Blood glucose concentration; BHI: Beverage hydration index; CHO: Carbohydrate; MEAL+S: Meal with Sustagen; NR: Not reported; ORS: Oral rehydration

solution; PPO: Peak power output; RH: Relative humidity; SD: Sports drink; TTE: Time to exhaustion; TT: Time trial; VO2max: Maximum oxygen uptake; W: Water.

Supplementary Material 6. Summary of studies exploring the effects of sodium bicarbonate on subsequent exercise performance and acid-base balance recovery after exhaustive exercise.

Study	Subject	VO_2max (ml.kg^{-1}.min^{-1})	Exercise Test	Recovery Period	Treatment	Supplementation timing	Result
**Gough et al. [103]**	9 active males	46.0 (±8.0)	Cycling TTE test at 100% PPO	90 min	**SBC:** 0.3 g.kg^{-1} $NaHO_3$<br><br>**PLA:** 0.1 g.kg^{-1} NaCl	30 min after 1st exercise	**TTE:** SBC < PLA<br>**RPE:** SBC = PLA<br>**Blood pH:** SBC > PLA<br>**[HCO_3^-]:** SBC > PLA<br>**[BLa^-]:** SBC > PLA
**Gough et al. [203]**	10 male trained cyclists	48.5 (±5.6)	2 × 4 km cycling TT in normobaric hypoxic chamber set at 14.5%, FiO_2 (~3000 m)	40 min	**SBC3:** 0.3 g.kg^{-1} $NaHO_3$<br><br>**SBC2:** 0.2 g.kg^{-1} $NaHO_3$<br><br>**PLA:** 0.07 g.kg^{-1} NaCl	180 min before 1st exercise	**TTE:** SBC3 = SBC2 = PLA<br>**RPE:** SBC3 = SBC2 = PLA<br>**[HCO_3^-]:** SBC3 < SBC2 < PLA<br>**SID:** SBC3 > SBC2 = PLA
**Gough et al. [204]**	7 elite male boxers	55.8 (±11.4)	Running HIIT, followed by running TTE test at 90% of VO_{2PEAK}	75 min	**SBC:** 0.3 g.kg^{-1} $NaHO_3$<br><br>**PLA:** 0.1 g.kg^{-1} NaCl	~10 min after 1st exercise	**TTE:** SBC < PLA<br>**RPE:** $NaHCO_3$ = PLA<br>**Blood pH:** SBC > PLA<br>**[HCO_3^-]:** SBC > PLA<br>**SID:** SBC > PLA
**Gurton et al. [205]**	11 recreational male runners	51.7 (±5.4)	Running TTE test at 100% vVO_2max	40 min	**SBC:** 0.3 g.kg^{-1} $NaHO_3$<br><br>**PLA:** 0.03 g.kg^{-1} NaCl	~5 min after 1st exercise	**TTE:** SBC = PLA<br>**RPE:** SBC = PLA<br>**Blood pH:** SBC = PLA<br>**[HCO_3^-]:** SBC = PLA

Abbreviations: [BLa^-]: Blood lactate concentration; [HCO_3^-]: Blood bicarbonate concentration; HIIT: High-intensity interval training; NR: Not reported; PLA: Placebo; PPO: Peak power output; RPE: Rate of perceived exertion; SBC: Sodium bicarbonate; SBC2: 0.2 g·kg^{-1} sodium bicarbonate; SBC3: 0.3 g·kg^{-1} sodium bicarbonate; SID: Strong ion difference ([K^+] + [Na^+] + [Ca^{2+}] + [Na^+] − [Cl^-] − [Lac^-]) from the end of the first exercise to the initial second exercise; TT: Time trial; TTE: Time to exhaustion; vVO_2max: Velocity associated with VO_2max.

Supplementary Material 7. Summary of studies exploring the effects of carbohydrate and creatine co-ingestion on subsequent exercise performance and glycogen resynthesis after exhaustive exercise.

Study	Subject	VO_2max (ml.kg^{-1}.min^{-1})	Exercise Test	Treatment	Duration of supplementation	Time of sampling	Result
**Robinson et al. [118]**	14 healthy men	NR	one-legged cycling TTE test at 160 to 170 bpm of heart rate	**CHO**: 4 times per day of (500 ml of CHO solution with 18.5% concentration) for 5 days<br><br>**CHO+CR**: 4 times per day of (500 ml of CHO solution with 18.5% concentration + 5 g.d^{-1} of creatine monohydrate) for 5 days	5 days of supplementation	After 6 h and day 5	**Total creatine**: CHO+CR in EXL < CHO+CR in NEXL > CHO in EXL and NEXL<br>**Muscle glycogen concentration**: CHO in EXL and NEXL = CHO+CR in EXL and NEXL
**Nelson et al. [126]**	12 physically active men	NR		**LOAD+CR**: 4 days of 6.6 g.kg^{-1}.d^{-1} of CHO in diet + 5 days of 20 g.d^{-1} of creatine monohydrate<br><br>**CR+LOAD**: 5 days of 20 g.d^{-1} of creatine monohydrate + 4 days of 6.6 g.kg^{-1}.d^{-1} of CHO in diet	4 days of CHO loading + 5 days of loading supplementation	Day 0 and 9	**Muscle glycogen concentration**: LOAD+CR < CR+LOAD
**Ööpik et al. [206]**	5 young healthy male wrestlers	NR	A maximal and submaximal isokinetic performance test on the Cybex II	**GLU**: 320 g of glucose<br><br>**GLU+CR**: 320 g of glucose + 30 g of creatine monohydrate	Single dose	-	**Body mass**: GLU = GLU+CR<br>Urine volume: GLU+CR = GLU<br>Maximal work: GLU+CR > GLU

							**BGC during exercise**: GLU+CR = GLU<br>*Strong correlation (r = 0.92) was observed between creatine retention and maximal work
**Van Loon et al. [125]**	20 young active males	62.4 (±1.6) and 63.8 (±2.2)	-	**CR**: 5 $g.day^{-1}$ for 5 days + 2 $g.day^{-1}$ for 42 days of creatine monohydrate supplementation<br><br>**PLA**: 5 $g.day^{-1}$ for 5 days + 2 $g.day^{-1}$ for 42 days of maltodextrin supplementation	5 days of loading + 6 weeks of continued supplementation	Day 0, 6 and 43	**Muscle glycogen concentration**: CR < PLA<br>**Insulin**: CR = PLA<br>**Total creatine**: CR < PLA<br>**Free creatine**: CR < PLA<br>**PCr**: CR < PLA<br>GLUT-4: CR = PLA<br>**mRNA expression of**:<br>• **GS-1**: CR + PLA<br>• **Gln-1**: CR + PLA
**Sewell et al. [207]**	6 healthy men	52.1 (±2.0)	TTE test on bicycle ergometer at 70% VO_2max	**CR**: 5 $g.day^{-1}$ of creatine for 5 days<br><br>**CHO**: 4 times per day of (500 ml of CHO solution with 18.5% concentration) for 5 days<br><br>**N**: Normal diet	5 days of supplementation	Before and immediately after exercise	**Body mass**: CR = CHO = N<br>**Muscle glycogen concentration at rest**: CHO < CR = N<br>**Muscle glycogen concentration after exercise**:

							CHO = CR = N<br>**Total creatine**: CR < CHO = N<br>**PCr**: CR = CHO = N<br>**ATP concentration at rest**: CR = CHO = N<br>**ATP concentration after exercise**: CR = CHO = N<br>**BGC during exercise**: CR = CHO = N
**Rico-Sanz [127]**	20 healthy trained males	NR	-	**CR+GLU:** 252 $g.day^{-1}$ of glucose + 21 $g.day^{-1}$ of creatine<br><br>**PLA+GLU:** 252 $g.day^{-1}$ of glucose + 21 $g.day^{-1}$ of maltodextrin	5 days	Day 0 and 5	**Muscle glycogen concentration**: CR+GLU = PLA+GLU<br>**Muscle PCr concentration**: CR+GLU < PLA+GLU
**Tomcik et al. [128]**	18 well-trained male cyclists and triathletes	65.10 (±7.1)	three performance trials of a 120 km cycling TT interspersed with alternating 1 and 4 km sprints (6 sprints each) performed every 10 km followed by an inclined TTE test at 90% VO_2peak.	**CR+MOD**: 20 $g.d^{-1}$ for 5 days + 3 $g.d^{-1}$ for 9 days of creatine monohydrate supplementation + 6 $g.kg^{-1}.d^{-1}$ of CHO in diet<br><br>**CR+LOAD**: 20 $g.d^{-1}$ for 5 days + 3 $g.d^{-1}$ for 9 days of creatine monohydrate supplementation + 12 $g.kg^{-1}.d^{-1}$ of CHO in diet<br><br>**PLA+MOD**: 20 $g.d^{-1}$ for 5 days + 3 $g.d^{-1}$ for 9 days of	5 days of loading + 9 days of continued supplementation	Day 0, 7 and 14	**TT**: CR+MOD = CR+LOAD = PLA+MOD = PLA+LOAD<br>**TTE**: CR+MOD = CR+LOAD = PLA+MOD = PLA+LOAD<br>**Body mass**: CR+MOD = CR+LOAD > PLA+MOD = PLA+LOAD<br>**Power of final 1 km sprint**: CR+MOD = CR+LOAD = PLA+MOD < PLA+LOAD<br>**Power of final 4 km sprint**: CR+MOD =

				placebo + 6 g.kg^{-1}.d^{-1} of CHO in diet<br>PLA-LOAD: 20 g.d^{-1} for 5 days + 3 g.d^{-1} for 9 days of placebo + 12 g.kg^{-1}.d^{-1} of CHO in diet		CR+LOAD > PLA+MOD = PLA+LOAD<br>**Total creatine**: CR+LOAD > PLA+LOAD<br>**Muscle glycogen concentration**: CR+LOAD > PLA+LOAD

Abbreviations: ATP: Adenosine triphosphate; CHO: Carbohydrate; CR+LOAD: Creatine supplementation followed by carbohydrate loading; CR+MOD: Creatine supplementation with a moderate carbohydrate diet; GLU: Glucose; GLUT-4: Glucose transporter type 4; Gln-1: Glycogenin-1; GS-1: Glycogen synthase-1; LOAD+CR: Carbohydrate loading followed by creatine supplementation; mRNA: Messenger RNA; NR: Not reported; PCr: Creatine phosphate; PLA+MOD: Placebo supplementation with a moderate carbohydrate diet; TT: Time trial; TTE: Time to exhaustion.

Supplementary Material 8. Summary of studies exploring the effects of caffeine and carbohydrate co-ingestion on muscle glycogen resynthesis after exhaustive exercise.

Study	Subject	VO_2max (ml.kg^{-1}.min^{-1})	Exercise Test	Treatment	Supplementation timing	Result
**Battram et al. [138]**	10 healthy males	60.9 (±2.4)	TTE test on bicycle ergometer at 75% of VO_2max	**CAFF:** pre-exercise (3 mg.kg^{-1}.h^{-1} caffeine) + during (3 mg.kg^{-1}.h^{-1} caffeine) + post-exercise (5 g.kg^{-1}.h^{-1} CHO)<br>**PLA:** pre-exercise (3 mg.kg^{-1}.h^{-1} placebo) + during (3 mg.kg^{-1}.h^{-1} placebo) + post-exercise (5 g.kg^{-1}.h^{-1} CHO)	Before and during exercise	CAFF = PLA
**Pedersen et al. [208]**	7 trained male cyclists and triathletes	60.0 (±3.7)	TTE test on bicycle ergometer at 70% VO_2peak	**CAFF:** 3 mg.kg^{-1}.h^{-1}<br>**CHO+CAFF:** 1 g.kg^{-1}.h^{-1} CHO + 2 mg.kg^{-1}.h^{-1} caffeine	After exercise	CHO+CAFF > CHO
**Beelen et al. [209]**	14 trained male cyclists	61.5 (±1.2)	Intermittent cycling test included 2 min of 90% to 70% PPO with 2 min active recovery at 50% PPO until exhaustion	**CHO:** 1.2 g.kg^{-1}.h^{-1} CHO<br>**CHO+PRO:** 1.2 g.kg^{-1}.h^{-1} CHO + 0.2 g.kg^{-1}.h^{-1} PRO hydrolysate + 0.1 g.kg^{-1}.h^{-1} leucine<br>**CHO+CAFF:** 1.2 g.kg^{-1}.h^{-1} CHO + 1.7 mg.kg^{-1}.h^{-1} caffeine	After exercise	CHO = CHO+PRO = CHO+CAFF

**Loureiro et al. [140]**	14 trained male cyclists and triathletes	59.9 (±8.3)	TTE test on bicycle ergometer at 70% PPO	**CAFF+MILK:** 1.2 $g.kg^{-1}.h^{-1}$ CHO + 0.3 $g.kg^{-1}.h^{-1}$ PRO + 2.7 $mg.kg^{-1}.h^{-1}$ caffeine + solid meal (sandwich) at 120 min<br><br>**MILK:** 1.2 $g.kg^{-1}.h^{-1}$ CHO + 0.3 $g.kg^{-1}.h^{-1}$ PRO + solid meal (sandwich) at 120 min	After exercise	CAFF+MILK > MILK

Abbreviations: CAFF: Caffeine; CHO: Carbohydrate; PLA: Placebo; PPO: Peak power output; PRO: Protein; TTE: Time to exhaustion; VO_2max: Maximum oxygen uptake during exercise; VO_2peak: Peak oxygen uptake during exercise.

Supplementary Material 9. Summary of studies exploring the effects of caffeine and carbohydrate co-ingestion on subsequent exercise performance.

Study	Subject	VO_2max (ml.kg^{-1}.min^{-1})	Pre-conditioning	Recovery Period	Treatment	Subsequent Exercise	Result
**Taylor et al. [210]**	6 recreationally active males	56 (±1)	A decreasing intermittent glycogen-depleting exercise protocol until volitional exhaustion	4 h	**CHO:** 1.2 g.kg^{-1}.h^{-1} CHO<br><br>**CHO+CAFF:** 1.2 g.kg^{-1}.h^{-1} CHO + 2 mg.kg^{-1}.h^{-1} caffeine<br><br>**PLA:** non-caloric flavored water	LIST	**TTE_{LIST}:** CHO+CAFF < CHO < PLA
**Andrade-Souza et al. [211]**	11 male amateur soccer players	43.9 (±2.3)	LIST	4 h	**CHO:** CHO 1.2 g.kg^{-1}.h^{-1}<br><br>**CHO+CAFF:** 1.2 g.kg^{-1}.h^{-1} CHO + 6 mg.kg^{-1} caffeine at 3 h after 1st exercise<br><br>**PLA:** non-caloric flavored water	LIST, LSPT, CMJ, RST	**TTE_{LIST}:** CHO+CAFF = CHO = PLA<br>**LSPT:** CHO+CAFF = CHO = PLA<br>**RST:** CHO+CAFF = CHO = PLA<br>**CMJ:** CHO+CAFF = CHO = PLA<br>**HR_{PEAK}:** CHO+CAFF = CHO = PLA<br>**[BLa^-]:** CHO+CAFF = CHO = PLA<br>**RPE:** CHO+CAFF = CHO = PLA
**Barzegar et al. [143]**	9 highly trained male paddlers	NR	Paddling 20 km TT	24 h	**CHO:** 1.2 g.kg^{-1}.h^{-1} CHO<br><br>**CHO+CAFF:** 1.2 g.kg^{-1}.h^{-1} CHO + 2 mg.kg^{-1}.h^{-1} caffeine	5 × on-water 500 m paddling TT with 3-min of active recovery	$TTE_{5×500}$: CHO+CAFF = CHO < PLA

	**PLA:** non-caloric flavored water	

Abbreviations: $[BLa^{-}]$: Blood lactate concentration; CAFF: Caffeine; CHO: Carbohydrate; CMJ: Countermovement jump; HR_{PEAK}: Peak heart rate; LIST: Loughborough Intermittent Shuttle Test; LSPT: Loughborough Soccer Passing Test; NR: Not reported; PLA: Placebo; RPE: Rate of perceived exertion; RST: Repeated sprint test; TT: Time trial; TTE: Time to exhaustion in Loughborough Intermittent Shuttle Test; $TTE_{5\times500}$: Time to exhaustion in 5 repetitions of 500 meters.
